# Metagenome reveals the midgut microbial community of *Haemaphysalis qinghaiensis* ticks collected from yaks and Tibetan sheep

**DOI:** 10.1186/s13071-024-06442-y

**Published:** 2024-08-31

**Authors:** Ying Zhang, Tian-Yin Cheng, Guo-Hua Liu, Lei Liu, De-Yong Duan

**Affiliations:** Research Center for Parasites & Vectors, College of Veterinary Medicine, Hunan Agricultural University, Changsha, 410128 Hunan Province China

**Keywords:** *Haemaphysalis qinghaiensis*, Microbial diversity, Microbiota, Metagenomics, Tick-borne pathogens, Ticks

## Abstract

**Background:**

*Haemaphysalis qinghaiensis* is a tick species distributed only in China. Due to its ability to transmit a variety of pathogens, including species of the genera *Anaplasma*, *Rickettsia*, *Babesia*, and *Theileria*, it seriously endangers livestock husbandry. However, the microbial community of the midgut of *H. qinghaiensis* females collected from yaks and Tibetan sheep has not yet been characterized using metagenomic sequencing technology.

**Methods:**

*Haemaphysalis qinghaiensis* were collected from the skins of yaks and Tibetan sheep in Gansu Province, China. Genomic DNA was extracted from the midguts and midgut contents of fully engorged *H. qinghaiensis* females collected from the two hosts. Metagenomic sequencing technology was used to analyze the microbial community of the two groups.

**Results:**

Fifty-seven phyla, 483 genera, and 755 species were identified in the two groups of samples. The ticks from the two hosts harbored common and unique microorganisms. At the phylum level, the dominant common phyla were Proteobacteria, Firmicutes, and Mucoromycota. At the genus level, the dominant common genera were *Anaplasma*, *Ehrlichia*, and *Pseudomonas*. At the species level, bacteria including *Anaplasma phagocytophilum*, *Ehrlichia minasensis*, and *Pseudomonas aeruginosa* along with eukaryotes such as *Synchytrium endobioticum* and *Rhizophagus irregularis*, and viruses such as the orf virus, *Alphadintovirus mayetiola*, and *Parasteatoda* house spider adintovirus were detected in both groups. In addition, the midgut of *H. qinghaiensis* collected from yaks had unique microbial taxa including two phyla, eight genera, and 23 species. Unique microorganisms in the midgut of *H. qinghaiensis* collected from Tibetan sheep included two phyla, 14 genera, and 32 species. Kyoto Encyclopedia of Genes and Genomes enrichment analysis revealed that the functional genes of the microbiome of *H. qinghaiensis* were annotated to six pathways, and the metabolic pathways included 11 metabolic processes, in which the genes involved in carbohydrate metabolism were the most abundant, followed by the genes involved in lipid metabolism.

**Conclusions:**

These findings indicate that most of the microbial species in the collected *H. qinghaiensis* ticks were the same in both hosts, but there were also slight differences. The analytical data from this study have enhanced our understanding of the midgut microbial composition of *H. qinghaiensis* collected from different hosts. The database of *H. qinghaiensis* microbe constructed from this study will lay the foundation for predicting tick-borne diseases. Furthermore, a comprehensive understanding of tick microbiomes will be useful for understanding vector competency and interactions with ticks and midgut microorganisms.

**Graphical abstract:**

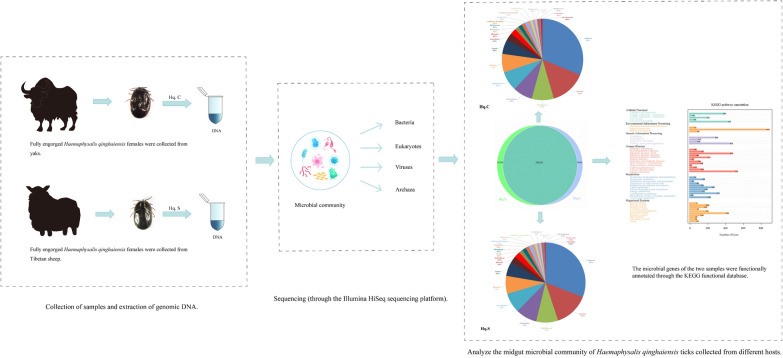

**Supplementary Information:**

The online version contains supplementary material available at 10.1186/s13071-024-06442-y.

## Background

*Haemaphysalis qinghaiensis* belongs to the family Ixodidae and the genus *Haemaphysalis* [1]. This tick species is distributed in Qinghai, Ningxia, Gansu, Sichuan, Yunnan, Tibet, and other western plateau areas of China [2]. Cattle, yaks, sheep, and goats are the main hosts of *H. qinghaiensis* [2], which is the main vector of theileriosis and piroplasmosis in sheep, goats, and yaks [3]. In addition to transmitting protozoa, a previous study has confirmed that *H. qinghaiensis* can also carry pathogens such as *Anaplasma ovis*, *Anaplasma bovis*, *Anaplasma phagocytophilum*, *Candidatus* Rickettsia tibetani, *Candidatus* Rickettsia gannanii, *Borrelia burgdorferi*, *Babesia motasi*-like, *Theileria sinensis*, *Theileria uilenbergi*, *Theileria luwenshuni*, and *Colpodella* spp. [4]. These pathogens were detected using polymerase chain reaction (PCR) assays, and there have been no studies using metagenomics technology to analyze the midgut microbial community of *H. qinghaiensis*. A comprehensive understanding of the viral, bacterial, eucaryotic, and archaean species in the midgut of *H. qinghaiensis* is essential for constructing a database of tick microbes which may lead to the ability to predict tick-borne diseases and to understand the relationship between microbes and their tick hosts.

Traditional microbial identification depends primarily on staining, culture, and biochemical tests. Although this method is still widely used, it is not suitable for microorganisms that cannot be cultured, especially for the microbiota of ticks, as most of the microbial species that ticks carry are still unknown. With the development of molecular technology, the PCR-denaturing gradient gel electrophoresis (PCR-DGGE) technique [5], FLX-titanium amplicon pyrosequencing [6], and high-throughput sequencing based on bacterial 16S ribosomal RNA (rRNA) V3–V4 regions [7] have been widely used in the analysis of the microbial communities of ticks. However, these studies have only analyzed and identified the bacterial communities in ticks and have not considered the viruses, eukaryotes, or archaea carried by ticks. Metagenomics is a microbial research method that directly examines the structure of all microbial communities and gene functions contained in samples. This method no longer depends on the isolation, culture, and purification of microorganisms, and it provides new ways to recognize and utilize more than 95% of uncultured microorganisms. To date, metagenomics has been applied in studies of the microbial communities of several tick species, including *Haemaphysalis longicornis* from cattle in Shanxi, China [8]; *Rhipicephalus microplus* from cattle in Hunan, China [9]; *Haemaphysalis japonica*, *Ixodes persulcatus*, *Haemaphysalis concinna*, and *Dermacentor silvarum* from northeastern China [10]; and *Ixodes ovatus*, *I. persulcatus*, and *Haemaphysalis flava* from mountainous areas of Shizuoka Prefecture, Japan [11]. The methodology has provided the foundation for a comprehensive understanding of the microbial species harbored by ticks.

In this study, the metagenomic approach was integrated to comprehensively analyze the midgut microbial community of *H. qinghaiensis* females collected from yaks and Tibetan sheep, and to determine the relative abundance of these microbial species. Subsequently, we also revealed the gene functions of the *H. qinghaiensis* microbiome by comparing unigenes with the Kyoto Encyclopedia of Genes and Genomes (KEGG) functional database. These findings may provide a comprehensive understanding of the microbial species in *H. qinghaiensis* females collected from different hosts, and show the respective metabolism of the *H. qinghaiensis* microbiome.

## Methods

### Collection of samples and extraction of genomic DNA

Fifty *H. qinghaiensis* at different engorged states were collected separately from the skins of yaks and Tibetan sheep in Lazikou Town, Diebu County, Gannan Tibetan Autonomous Prefecture, Gansu Province, China (34°14′ N, 103°52′ E). All ticks were immediately sent to the Parasite and Vector Research Center of Hunan Agricultural University College of Veterinary Medicine, Changsha City, Hunan Province, China. After weighing, 0.2250 g ± 0.005 g was used as the standard to define fully engorged female ticks. Five fully engorged *H. qinghaiensis* females collected from different yaks and five fully engorged *H. qinghaiensis* females collected from different Tibetan sheep were used in this study. Before dissection of ticks, all dissecting instruments, plasticware, glassware, buffers (including phosphate-buffered saline, PBS), and solutions used in this study were sterilized by autoclaving and ultraviolet (UV) light treatment. All experimental operations were conducted on an ultra-clean workbench with UV sterilization to protect the samples from environmental pollution.

The two groups of female ticks collected from different hosts were surface-disinfected with 75% (v/v) alcohol for 2 min, and then the surface of each tick was disinfected with 5% (v/v) NaClO solution. The two groups of ticks were washed with sterilized water three times to remove the disinfectant and any impurities. The integument of the tick was cut along the abdomen using sterilized ophthalmic scissors. The midguts and midgut contents of fully engorged *H. qinghaiensis* females collected from yaks and Tibetan sheep were collected into two 1.5 ml sterile centrifuge tubes containing 1 ml of 3.8% sodium citrate sterile saline solution. Then the two tubes were centrifuged at 55×*g* for 10 min. After centrifugation, the supernatants of the two samples were taken into two new 1.5 ml sterile centrifuge tubes and centrifuged at 8609×*g* for 1 min. After centrifugation, the precipitates were retained and labeled the samples of ticks collected from yaks and Tibetan sheep as Hq. C and Hq. S, respectively.

For each sample group, 1000 μl of hexadecyltrimethylammonium bromide lysate (CTAB lysate) and 20 μl of lysozyme were added to the precipitates and mixed well. The samples were placed in a water bath at 65 °C for 2 h, during which the tubes were inverted several times to ensure that the samples were fully lysed. After centrifuging at 5022×*g* for 10 min, 950 μl of supernatant of each sample was retained. An equal volume of phenol (pH 8.0)/chloroform/isoamyl alcohol (25:24:1) was added to the supernatant of each sample and mixed while inverted. After centrifuging at 8609×*g* for 10 min, the supernatant of each sample was retained, and the same volume of chloroform/isoamyl alcohol (24:1) was added and mixed while inverted. After centrifuging at 8609×*g* for 10 min, the supernatant of each sample was taken into a 1.5 ml centrifuge tube. Then, a 3/4 volume of isopropanol was added to the supernatant of each sample, and the tubes were shaken for mixing. After centrifuging at 8609×*g* for 10 min, the liquid was removed, and the precipitate of each sample was washed twice with 1 ml of 75% (v/v) ethanol. After extracting the alcohol from each centrifuge tube, the precipitate of each sample was dried at room temperature. Then, 51 μl of double-distilled water (ddH_2_O) was added to the precipitate of each sample to dissolve the DNA, after which 1 μl of RNase A was added. The samples were then incubated at 37 °C for 15 min to digest the RNA. Electrophoresis using 1% agarose gel was used to detect the purity and integrity of the DNA in each sample. The genomic DNA of each sample was quantified using a Qubit^®^ dsDNA Assay Kit (Thermo Fisher Scientific, Waltham, MA, USA) for the Qubit^®^ 2.0 Fluorometer (Thermo Fisher Scientific).

### Library construction and sequencing

A total of 1 μg of DNA per sample was used for constructing sequencing libraries. The sequencing libraries were generated using a NEBNext Ultra DNA Library Prep Kit (New England Biolabs, Ipswich, MA, USA) following the manufacturer’s protocol. After the sequencing library construction was completed, Qubit^®^ 2.0 (Thermo Fisher Scientific, Waltham, MA, USA) was used for preliminary quantification. The insert size of the libraries was measured using an Agilent 2100 Bioanalyzer (Agilent Technologies, Palo Alto, CA, USA). If the insert size followed expectations, the effective concentration (> 3 nM) of the libraries was quantified using real-time PCR (Thermo Fisher Scientific, Waltham, MA, USA). After passing each library test, mixed sequencing was performed according to the requirements of effective concentration and 10,240 million base pairs (Mbp) offline data volume, followed by Illumina HiSeq paired-end (PE)150 sequencing.

### Sequencing data processing and metagenome assembly

Readfq software (v8, https://github.com/cjfields/readfq) was used to remove reads with low-quality bases (the default quality threshold was less than 38) exceeding a certain proportion (the default length value was 40 bp) from the raw data obtained from the Illumina HiSeq sequencing platform, remove reads with N bases reaching a certain proportion (the default length value was 10 bp), and remove reads with overlap with the adapter exceeding a certain threshold (the default length value was 15 bp). Afterward, the processed reads were compared with the host database using Bowtie2 software (v2.2.4, http://bowtie-bio.sourceforge.net/bowtie2/index.shtml) (the parameter options were -end-to-end, -sensitive, -*I* 200, *-X* 400) [12] to filter out reads from the host and obtain clean data. The clean data were assembled using MEGAHIT software (v1.0.4-beta) [the parameter options were -presets meta-large (-end-to-end, -sensitive, -*I* 200, -*X* 400)] [13]. The assembled scaffolds were interrupted from the N junction to obtain scaftigs without N bases [14].

### Gene prediction and abundance analysis

MetaGeneMark software (v2.10, http://topaz.gatech.edu/GeneMark/) was used to predict the open reading frames of the scaftigs (≥ 500 bp) of each sample [15]. Sequence information from the predicted results with lengths of less than 100 nucleotides was filtered [14]. A non-redundant gene catalogue was constructed with CD-HIT software (v4.5.8, http://www.bioinformatics.org/cd-hit/) [16] (the parameter options were -c 0.95, -G 0, -aS 0.9, -g 1, -d 0) [17] using a sequence identity cut-off of 0.95, with a minimum coverage cut-off of 0.9 for the shorter sequences. Bowtie2.2.4 (the parameter settings were -end-to-end, -sensitive, -*I* 200, -*X* 400) [12] was used to map the clean data of each sample to the gene catalogue to obtain the reads to which genes were mapped in each sample. Then, the genes for which the number of reads was ≤ 2 in each sample were filtered to obtain unigenes [18]. The abundance of information for each gene in each sample was calculated based on the number of mapped reads and the lengths of the genes. The measurement unit used in this study was transcripts per million (TPM). The formula is

$${G}_{k}=\frac{{r}_{k}}{{L}_{k}}\cdot \frac{1}{{\sum }_{i=1}^{n}\frac{{r}_{i}}{{L}_{i}}},$$

where *r* is the number of mapped reads, and *L* is the lengths of the genes.

### Species annotation

DIAMOND software (v0.9.9.110, https://github.com/bbuchfink/diamond/) [the parameter settings were Basic Local Alignment Search Tool Program (BLASTP) E value ≤ 1e−5] [19] was used to compare the unigenes with the sequences of bacteria, eukaryotes, archaea, and viruses that were extracted from the NR database (V2018-01-02, https://www.ncbi.nlm.nih.gov/) of the National Center for Biotechnology Information (NCBI). The results having an E value of ≤ 1e−10 were selected to determine the species annotation information by the latent class analysis algorithm that was applied to the systematic classification from MEGAN software [20] to verify the species annotation information of the sequences [21]. According to the latent class analysis annotation results and the gene abundance information, relative abundance information tables and the gene number tables of each sample at different taxonomic hierarchies (boundary, phylum, class, order, family, genus, and species) were obtained. Krona analysis, relative abundance profile analysis, and the construction of an abundance cluster heat map were performed based on the abundance table for each taxonomic hierarchy.

### Common function database annotations

DIAMOND software (v0.9.9.110, https://github.com/bbuchfink/diamond/) (the parameter settings were BLASTP, E value ≤ 1e−5) [19] was used to compare the unigenes with the KEGG database (V2018-01-01, http://www.kegg.jp/kegg/) [22]. For each sequence alignment result, the best blast hit (one high-scoring segment pair with more than 60 bits) was used for subsequent analysis [23]. The relative abundance of different functional levels was calculated based on the comparison results. A table of the number of genes at each classification level of the two groups was obtained based on the results of functional annotation and the relative abundance of genes. According to the relative abundance table for each classification level, the statistics of the number of annotated genes were obtained; a relative abundance profile was constructed, and comparative analyses of metabolic pathways were performed.

## Results

### DNA quality

A total of 12,757.12 Mbp of clean data were generated by sequencing using the Illumina HiSeq sequencing platform. The effective data rate of the two sample groups was 99.0%. Specific data output statistics and quality control information are shown in Table 1.

**Table 1:** Quality parameters of the sequencing data for the DNA of each sample

Sample^a^	Insert size^b^(bp)	Raw data^c^(Mbp)	Clean data^d^(Mbp)	Clean Q20^e^	Clean Q30^f^	Clean GC^g^(%)	Effective^h^(%)
Hq. C	350	6336.22	6274.58	96.03	90.75	47.45	99.027
Hq. S	350	6535.33	6482.54	96.31	91.41	47.80	99.192

^a^Sample name

^b^Insert length (default 350 bp library)

^c^Raw data from the computer

^d^Clean data obtained by filtering

^e^Sequencing error rate in clean data is < 0.01 (quality is the percentage of bases with a value > 20)

^f^Sequencing error rate in clean data is < 0.001 (quality is the percentage of bases with a value > 30)

^g^GC content of bases in clean data

^h^Percentage of clean data and raw data

### General statistics

Following quality control, 118,170 and 119,566 genes were obtained from the sequencing data of Hq. C and Hq. S, respectively. There were 108,182 genes common to the two groups. The numbers of genes specific to Hq. C and Hq. S were 9988 and 11,384, respectively. The results showed that most of the microbial populations in the midgut of fully engorged *H. qinghaiensis* females collected from yaks and Tibetan sheep were the same, but there were also slight differences between the two groups.

### Relative abundance of microorganisms

**Fig. 1:**
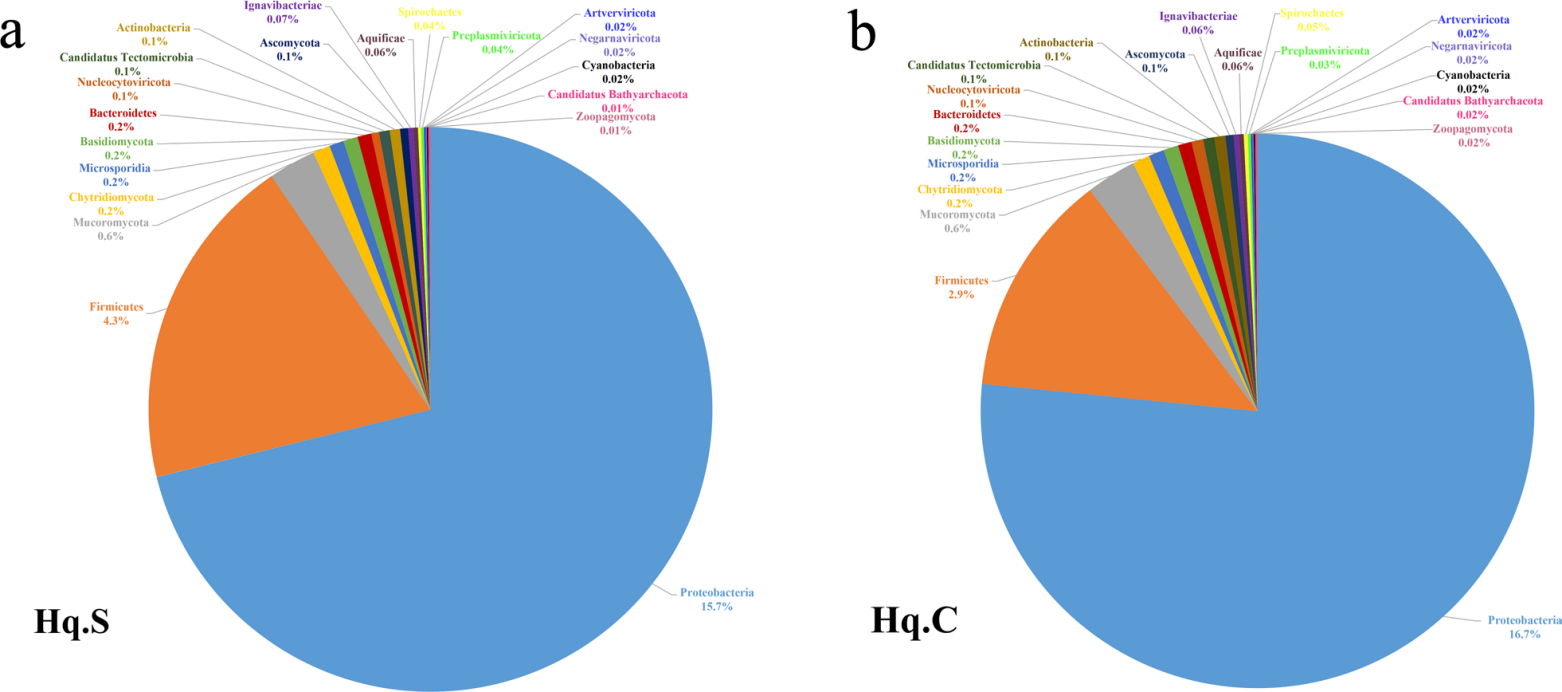
Microbial population characteristics of the top 20 common phyla in the midgut of fully engorged *Haemaphysalis qinghaiensis* females collected from Tibetan sheep (**a**) and yaks (**b**)

At the phylum level, a total of 57 phyla were identified in the samples, and 53 phyla were common to both groups. Among the common phyla, there were 29 bacterial phyla, eight viral phyla, nine eukaryotic phyla, and seven archaean phyla. The distribution characteristics of the top 20 common phyla in the two groups are shown in Fig. 1a, b, and the relative abundance values for the remaining common phyla are listed in Additional file 1: Table S1. In the common phyla, Proteobacteria, Firmicutes, Mucoromycota, Chytridiomycota, Microsporidia, Basidiomycota, Bacteroidetes, Nucleocytoviricota, Candidatus Tectomicrobia, Actinobacteria, and Ascomycota had the highest relative abundance. The relative abundance of Proteobacteria in Hq. C and Hq. S was 16.7% and 15.7%, respectively, and this was the dominant phylum for both groups. Firmicutes (Hq. C 2.9% and Hq. S 4.3%) had lower relative abundance. In addition, Balneolaeota and Candidatus Giovannonibacteria only existed in Hq. C, and candidate division Zixibacteria and Candidatus Aerophobetes only existed in Hq. S.

**Fig. 2:**
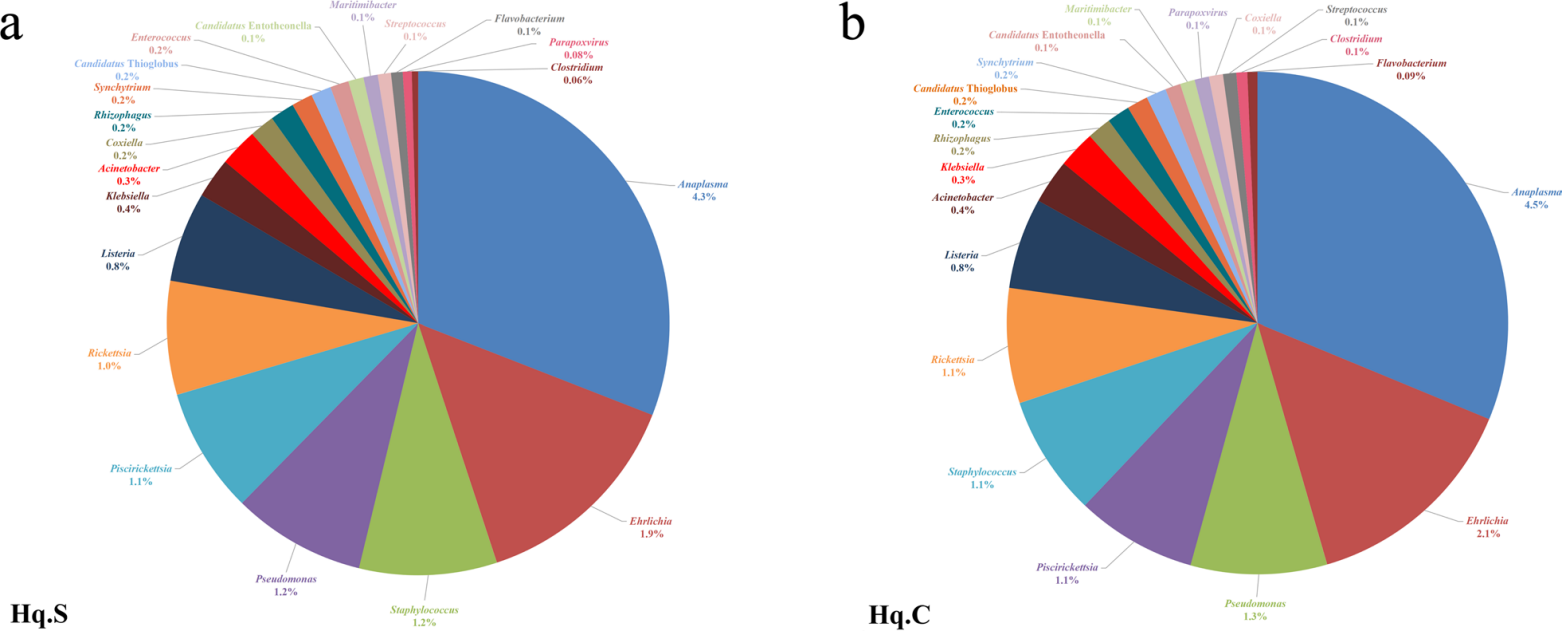
Microbial population characteristics of the top 20 common genera in the midgut of fully engorged *Haemaphysalis qinghaiensis* females collected from Tibetan sheep (**a**) and yaks (**b**)

At the genus level, a total of 483 genera were identified in the two groups, of which 461 genera were common to both groups. For the common genera, there were 222 bacterial genera, 21 viral genera, 212 eukaryotic genera, and six archaean genera. The distribution characteristics of the top 20 common genera in the two groups are shown in Fig. 2a, b, and the relative abundance values for the remaining common genera are listed in Additional file 2: Table S2. For the common genera, *Anaplasma*, *Ehrlichia*, *Pseudomonas*, *Staphylococcus*, *Piscirickettsia*, *Rickettsia*, *Listeria*, *Acinetobacter*, *Klebsiella*, *Rhizophagus*, *Coxiella*, *Enterococcus*, *Candidatus* Thioglobus, and *Synchytrium* had the highest relative abundance. The relative abundance of *Anaplasma* in Hq. C and Hq. S was 4.5% and 4.3%, respectively, and this was the dominant genus in both groups. *Ehrlichia* (Hq. C 2.1% and Hq. S 1.9%) had lower relative abundance. The relative abundance of *Rickettsia*, *Piscirickettsia*, *Staphylococcus*, and *Pseudomonas* in the two groups was 1–1.3%. In addition to the common genera, unique genera were present in the two groups, and the relative abundance values for the unique genera in each group are shown in Table 2.

**Table 2:** Relative abundance of unique genera in the midgut of fully engorged *Haemaphysalis qinghaiensis* females collected from yaks (Hq. C) or Tibetan sheep (Hq. S)

Kingdom	Genus	Abundance (%)	
		Hq. C	Hq. S
Bacteria	*Kallotenue*	0.00000173	0
	*Olivibacter*	0.00000137	0
	*Niastella*	0.00000136	0
	*Facklamia*	0.00000126	0
	*Fredinandcohnia*	0.00000086	0
	*Arthrobacter*	0	0.00000614
	*Solirubrobacter*	0	0.00000399
	*Frankia*	0	0.00000237
	*Bifidobacterium*	0	0.00000190
	*Henriciella*	0	0.00000183
	*Lysinibacillus*	0	0.00000104
	*Eudoraea*	0	0.00000099
	*Photobacterium*	0	0.00000081
	*Modestobacter*	0	0.00000078
	*Persephonella*	0	0.00000062
Eukaryota	*Hypoxylon*	0.00000210	0
	*Filobasidium*	0.00000205	0
	*Zygosaccharomyces*	0.00000089	0
	*Hyphodiscus*	0	0.00000123
	*Yarrowia*	0	0.00000083
	*Tetrapisispora*	0	0.00000082
Viruses	Ascovirus	0	0.00000151

**Fig. 3:**
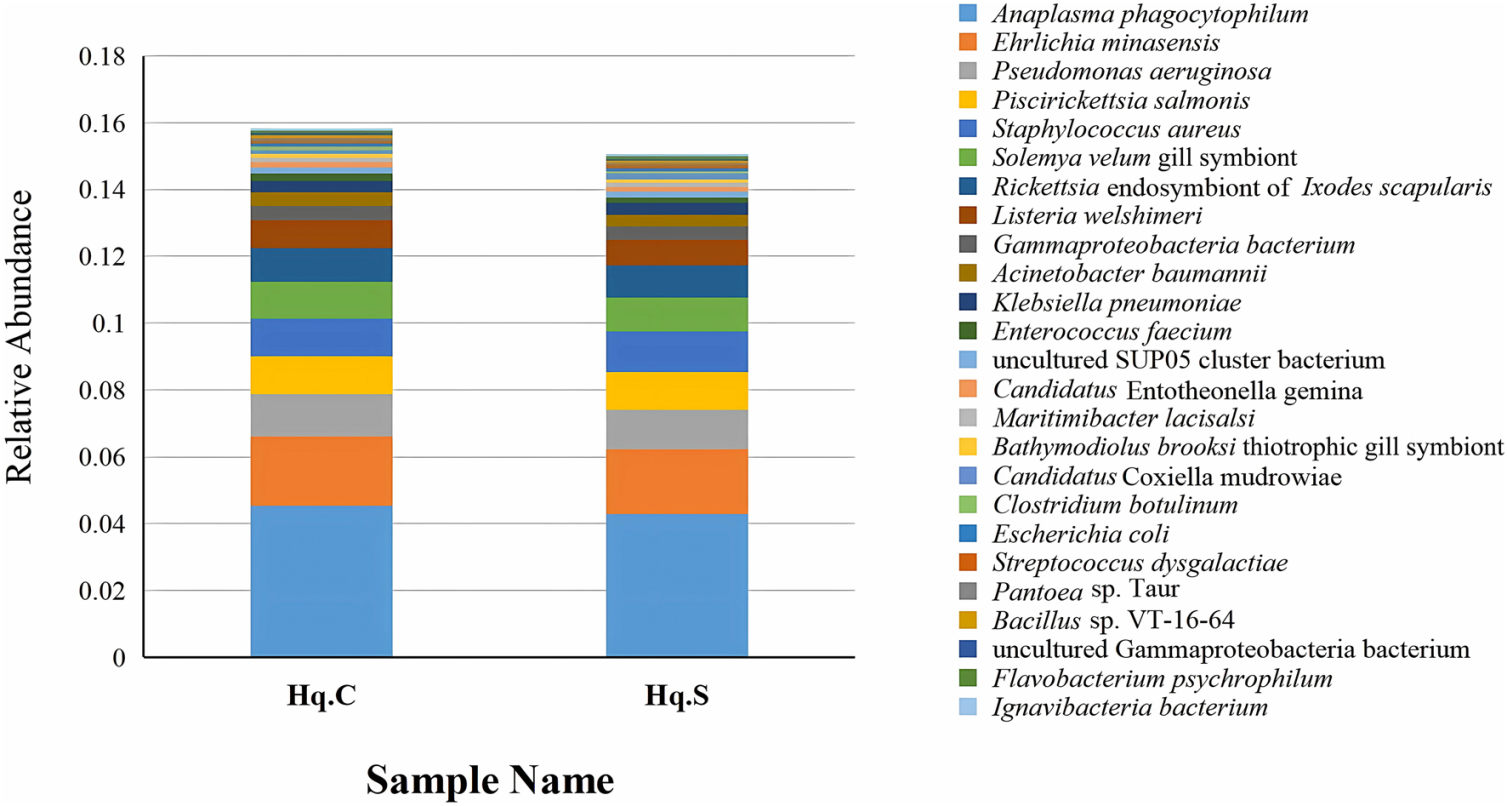
Microbial population characteristics of the top 25 common bacterial species in the midgut of fully engorged *Haemaphysalis qinghaiensis* females collected from yaks (Hq. C) and Tibetan sheep (Hq. S)

**Fig. 4:**
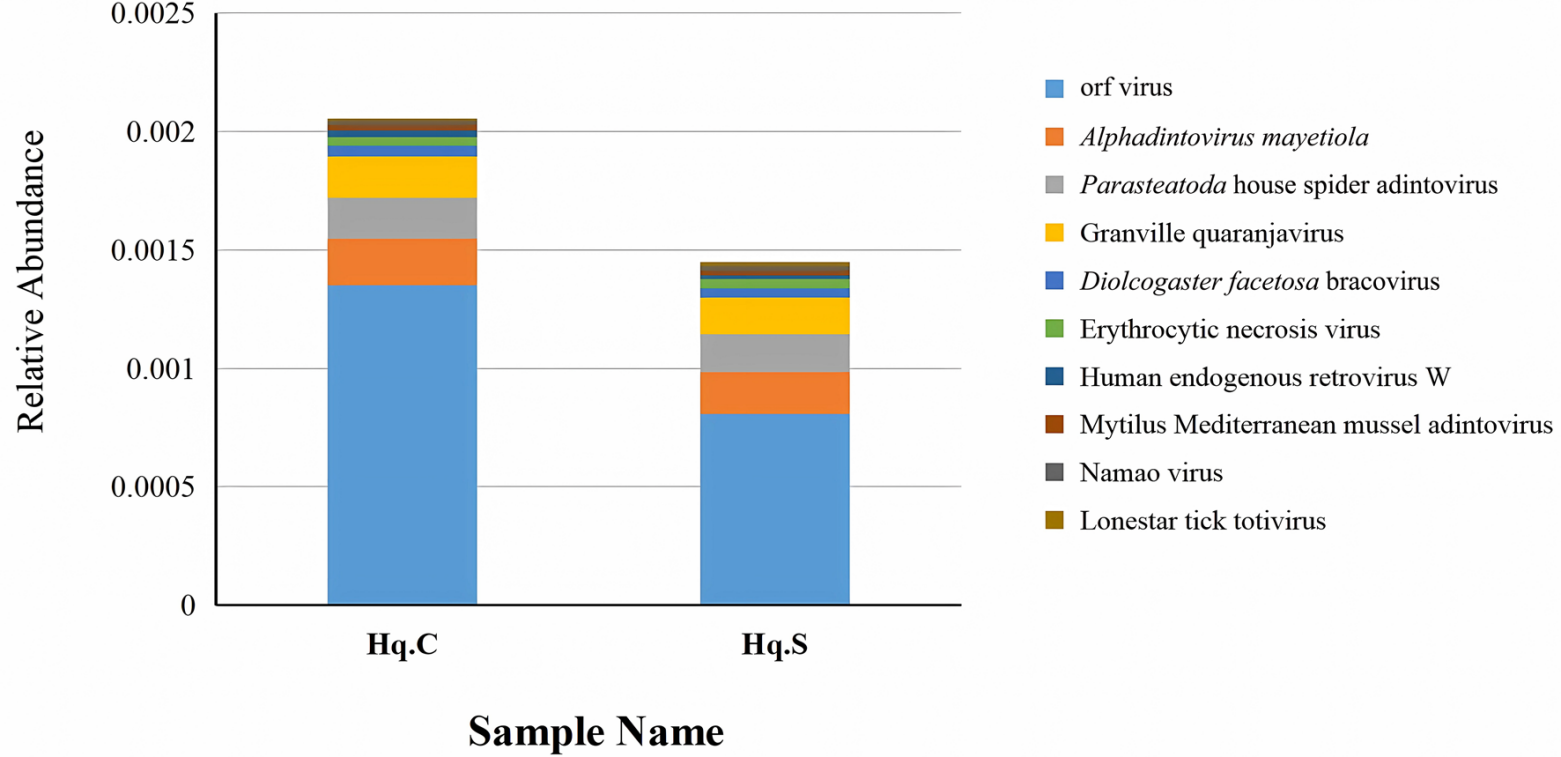
Microbial population characteristics of the top 10 common viral species in the midgut of fully engorged *Haemaphysalis qinghaiensis* females collected from yaks (Hq. C) and Tibetan sheep (Hq. S)

At the species level, a total of 755 species were identified in the two groups, of which 700 species were common to both groups. Among the common species, there were 341 bacterial species, 61 viral species, 285 eukaryotic species, and 13 archaean species. The distribution characteristics of the top 25 common bacterial species are shown in Fig. 3, and the relative abundance values for the remaining common bacterial species are listed in Additional file 3: Table S3. The distribution characteristics of the top 10 common viral species in the two groups are shown in Fig. 4, and the relative abundance values for the remaining common viral species are listed in Additional file 4: Table S4. The relative abundance values for the common eukaryotic and archaean species are listed in Additional file 5: Table S5 and Additional file 6: Table S6, respectively.

In the two groups, *A. phagocytophilum*, *Ehrlichia minasensis*, *Pseudomonas aeruginosa*, *Staphylococcus aureus*, *Piscirickettsia salmonis*, *Solemya velum* gill symbiont, and *Rickettsia* endosymbiont had the highest relative abundance and were the dominant bacterial species. In particular, *Anaplasma* was identified as *A. phagocytophilum*, and the relative abundance of *A. phagocytophilum* in Hq. C and Hq. S was 4.5% and 4.3%, respectively, indicating significant dominance. The relative abundance was equal between the two groups for *A. phagocytophilum* and the genus *Anaplasma*, suggesting that only the bacterial species of *A. phagocytophilum* in the genus *Anaplasma* existed in the midgut of fully engorged *H. qinghaiensis* females collected from yaks and Tibetan sheep. *Synchytrium endobioticum*, *Rhizophagus irregularis*, *Dictyocoela muelleri*, *Cucumispora dikerogammari*, *Nosema granulosis*, and *Mucor circinatus* were the common eukaryotic species that had the highest relative abundance in the two groups. The dominant viruses were orf virus, *Alphadintovirus mayetiola*, *Parasteatoda* house spider adintovirus, and Granville quaranjavirus. *Candidatus* Bathyarchaeota archaeon and *Candidatus* Heimdallarchaeota archaeon B3_Heimare were the common archaean species with high relative abundance in both groups. In addition to the common microbial species, unique species were also present in both groups. Twenty-three microbial species were unique to Hq. C, of which 15 were bacteria, four were eukaryotes, and four were viruses. The relative abundance of each unique microorganism is shown in Table 3. Thirty-two microbial species were unique to Hq. S, of which 20 were bacteria, four were eukaryotes, and eight were viruses. The relative abundance values for each unique microorganism are listed in Table 4.

**Table 3:** Relative abundance of unique species in the midgut of fully engorged *Haemaphysalis qinghaiensis* females collected from yaks

Kingdom	Species	Abundance (%)	
		Hq. C	Hq. S
Bacteria	*Clostridium paraputrificum*	0.00000417	0
	*Dehalococcoidia* bacterium	0.00000273	0
	*Kallotenue* sp. CFH 73958	0.00000173	0
	*Pseudoalteromonas* sp. BMB	0.00000166	0
	*Olivibacter* sp. XZL3	0.00000137	0
	*Wolbachia* endosymbiont	0.00000137	0
	*Bryobacterales* bacterium F-183	0.00000136	0
	*Niastella* sp. SYSU D00799	0.00000136	0
	*Gemmatimonadales* bacterium	0.00000130	0
	*Facklamia hominis*	0.00000126	0
	*Cyclobacterium xiamenense*	0.00000113	0
	*Candidatus* Giovannonibacteria bacterium RIFCSPHIGHO2_01_FULL_48_47	0.00000109	0
	*Fredinandcohnia aciditolerans*	0.00000086	0
	*Vibrio marinisediminis*	0.00000082	0
	*Kitasatospora aureofaciens*	0.00000071	0
Eukaryota	*Hypoxylon* sp. CI-4A	0.00000210	0
	*Filobasidium floriforme*	0.00000205	0
	*Metarhizium majus*	0.00000176	0
	*Acaulospora colombiana*	0.00000071	0
Viruses	Enzootic nasal tumor virus of goats	0.00000947	0
	Jaagsiekte sheep retrovirus	0.00000902	0
	Norway mononegavirus 1	0.00000122	0
	*Diatraea saccharalis* granulovirus	0.00000119	0

**Table 4:** Relative abundance of unique species in the midgut of fully engorged *Haemaphysalis qinghaiensis* females collected from Tibetan sheep

Kingdom	Species	Abundance (%)	
		Hq. C	Hq. S
Bacteria	*Vibrio ouci*	0	0.00001250
	*Pseudomonas protegens*	0	0.00000767
	*Arthrobacter* sp. M6	0	0.00000614
	*Streptomyces plicatus*	0	0.00000502
	*Solirubrobacter* sp. CPCC 204708	0	0.00000399
	*Nocardia blacklockiae*	0	0.00000335
	*Klebsiella variicola*	0	0.00000188
	*Henriciella aquimarina*	0	0.00000183
	*Pseudomonas* sp. 32_A	0	0.00000168
	*Candidatus* Frankia datiscae	0	0.00000156
	Candidate division Zixibacteria bacterium	0	0.00000143
	*Pseudomonas* sp. MAP12	0	0.00000126
	*Lysinibacillus sphaericus*	0	0.00000104
	*Pseudomonas tolaasii*	0	0.00000103
	Marine gamma proteobacterium HTCC2080	0	0.00000103
	*Candidatus* Aerophobetes bacterium ADurb.Bin490	0	0.00000102
	*Eudoraea adriatica*	0	0.00000099
	*Photobacterium alginatilyticum*	0	0.00000081
	*Modestobacter lapidis*	0	0.00000078
	*Persephonella* sp.	0	0.00000062
Eukaryota	*Hyphodiscus hymeniophilus*	0	0.00000123
	*Malassezia sympodialis*	0	0.00000104
	*Yarrowia* sp. E02	0	0.00000083
	*Tetrapisispora blattae*	0	0.00000082
Viruses	Bat gammaretrovirus	0	0.00005640
	*Odocoileus hemionus* endogenous retrovirus	0	0.00000353
	*Pipistrellus pipistrellus* endogenous retrovirus	0	0.00000202
	Ovine enzootic nasal tumor virus	0	0.00000201
	Gibbon ape leukemia virus	0	0.00000164
	*Myotis daubentonii* endogenous retrovirus	0	0.00000153
	Prosimian retrovirus 1	0	0.00000151
	Hubei diptera virus 17	0	0.00000058

### Cluster analysis of the relative abundance of the two sample groups

**Fig. 5:**
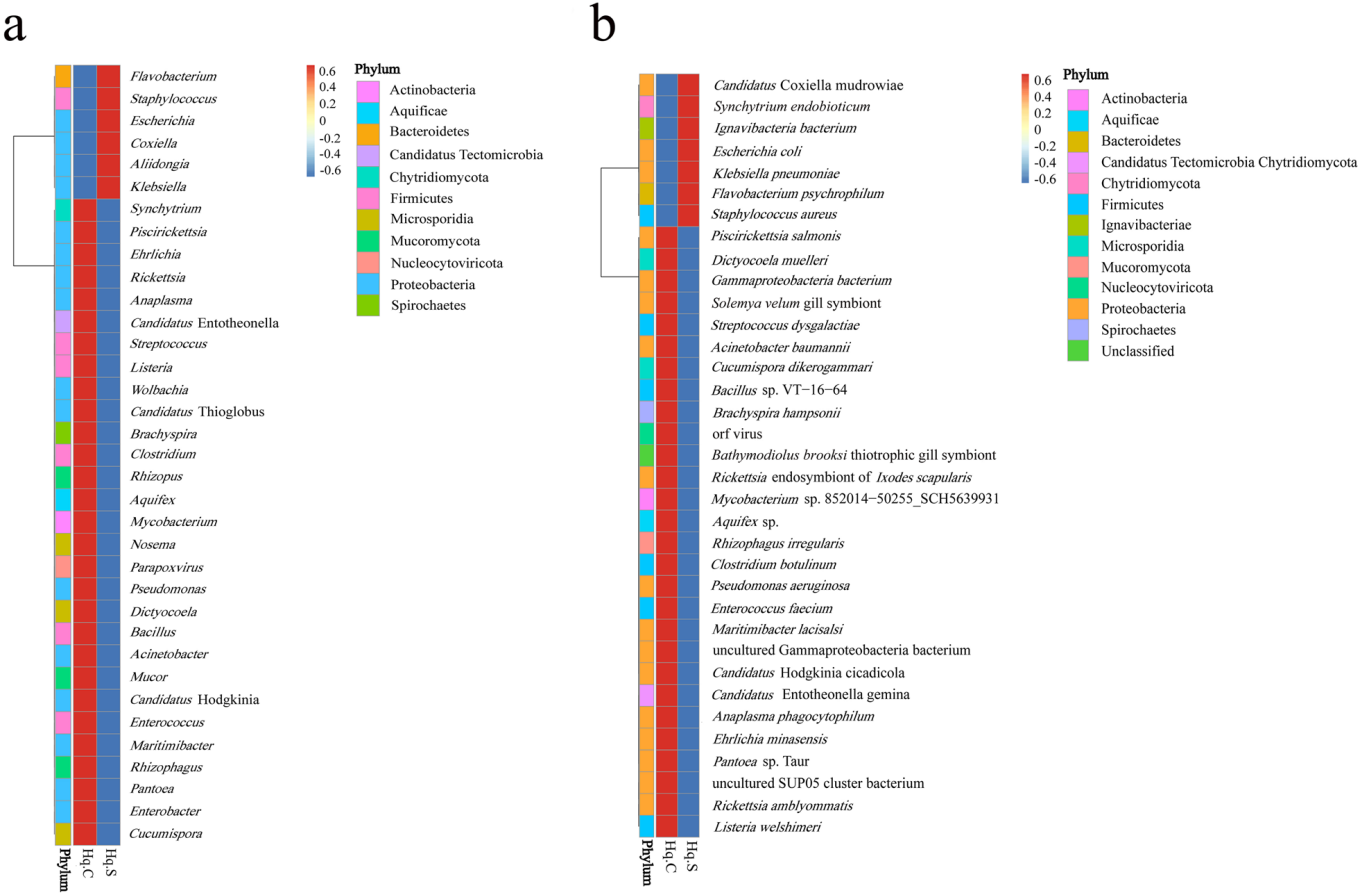
Cluster analysis of the relative abundance of the two sample groups at genus (**a**) and species (**b**) level. The data are represented by different colors in the heatmap, which represent the score calculated using the relative abundance

The 35 most common genera and their abundance information for each sample were selected to draw a heatmap (Fig. 5a). Six genera had low relative abundance in the sample of ticks collected from yaks but high relative abundance in the sample of ticks collected from Tibetan sheep. These six genera included *Flavobacterium*, *Staphylococcus*, *Escherichia*, *Coxiella*, *Aliidongia*, and *Klebsiella.* The remaining 29 genera had high relative abundance in the sample of ticks collected from yaks but low in the sample of ticks collected from Tibetan sheep.

The 35 most common species and their abundance information for each sample were used to draw a heatmap (Fig. 5b). Seven species had low relative abundance in the sample of ticks collected from yaks but high relative abundance in the sample of ticks collected from Tibetan sheep. The seven species included *Candidatus* Coxiella mudrowiae, *Synchytrium endobioticum*, *Ignavibacteria bacterium*, *Escherichia coli*, *Klebsiella pneumoniae*, *Flavobacterium psychrophilum*, and *S. aureus.* The remaining 28 species had high relative abundance in the sample of ticks collected from yaks but low relative abundance in the sample of ticks collected from Tibetan sheep.

### Gene function prediction for the microbiome

**Fig. 6:**
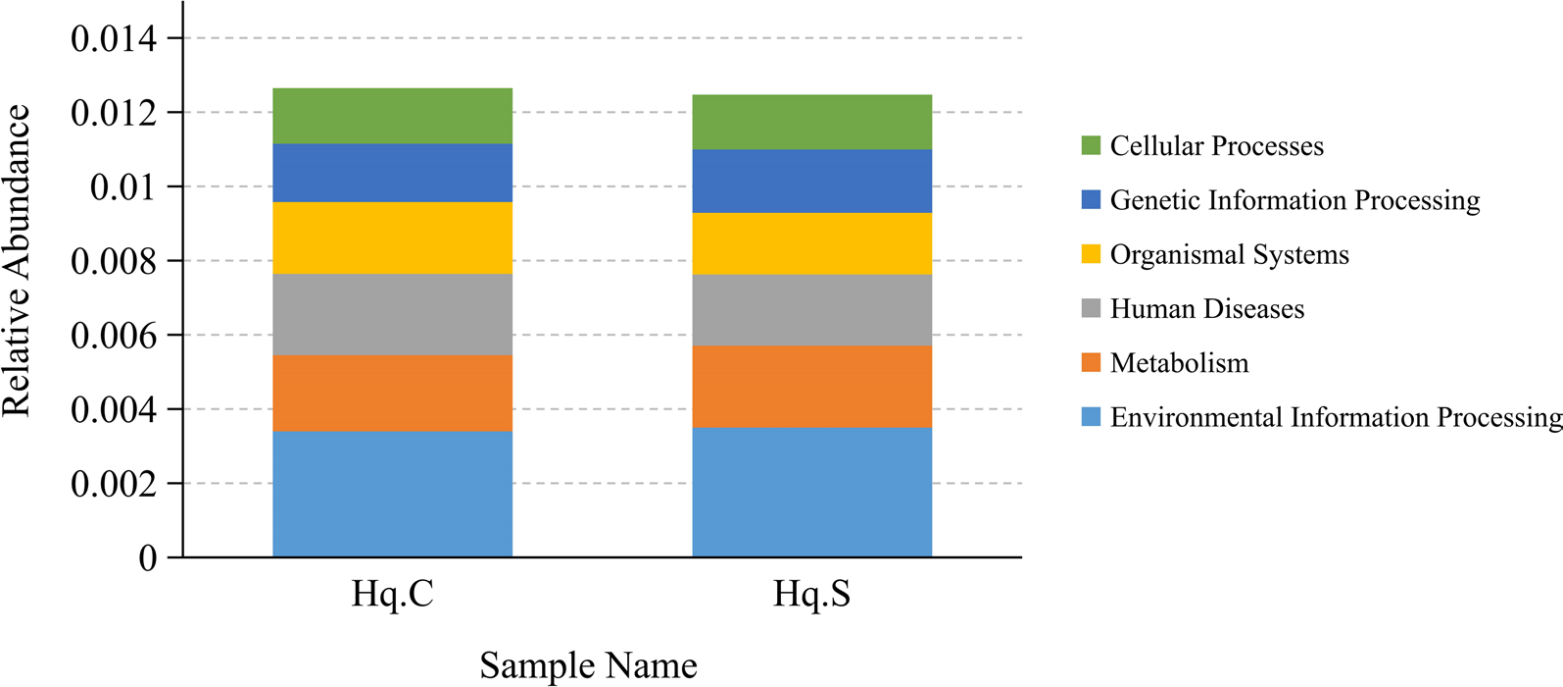
Relative abundance of six KEGG pathways of the midgut microbiome in the two groups of *Haemaphysalis qinghaiensis* females collected from yaks (Hq. C) and Tibetan sheep (Hq. S) at level 1

**Fig. 7:**
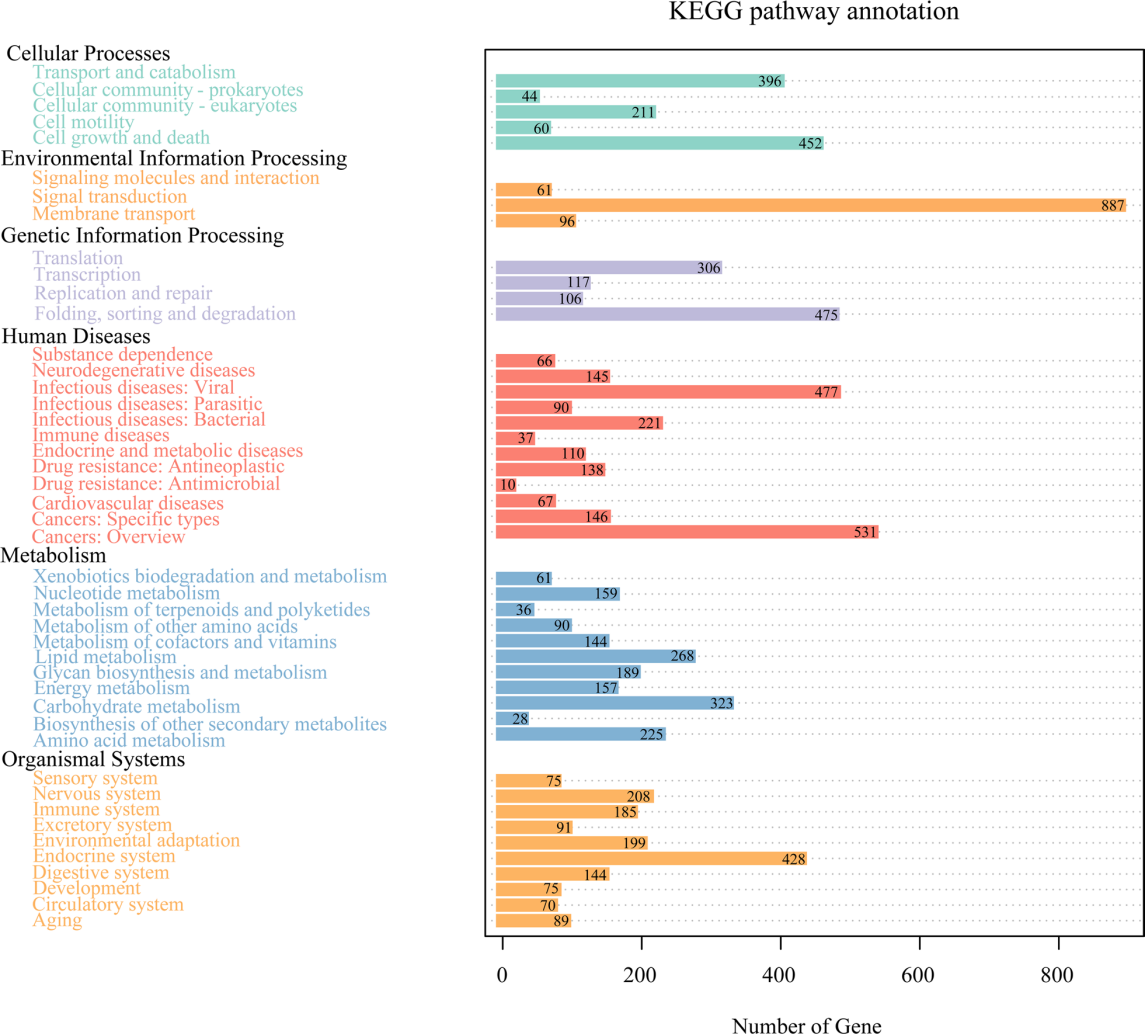
Annotations of KEGG pathways related to the number of genes of the midgut microbiome in the two groups of *Haemaphysalis qinghaiensis* females collected from different hosts. The black fonts on the ordinate indicate KEGG level 1, the colored fonts indicate the specific pathway at level 2, and the abscissa indicates the number of genes in the pathway

DIAMOND software was used to compare the unigenes with the KEGG database. The relative abundance of different functional levels was measured based on the alignment results. At level 1, the functional genes of the microbiome of *H. qinghaiensis* were annotated to six KEGG pathways: Environmental Information Processing, Metabolism, Human Diseases, Organismal Systems, Genetic Information Processing, and Cellular Processes. The differences in the relative abundance of each pathway between the two groups were relatively small (Fig. 6). At level 1, 1680 functional genes were annotated to metabolic pathways in the microbiome of *H. qinghaiensis*, while 2038 functional genes were annotated to human disease pathways. At level 2, the functional genes in the *H. qinghaiensis* microbiome were associated with 11 metabolic processes, with the functional genes involved in carbohydrate metabolism being the most abundant, followed by the genes involved in lipid metabolism (Fig. 7). In human disease pathways, the genes associated with infectious diseases (e.g., *Salmonella* infection, pathogenic *Escherichia coli* infection, herpes simplex infection, and Epstein–Barr virus infection) were more abundant.

## Discussion

This study reveals and analyzes the characteristics of the midgut microbiome composition of fully engorged *H. qinghaiensis* females collected from yaks and Tibetan sheep using metagenomics technology. We identified 57 phyla, 483 genera, and 755 species in the two groups of samples. There were both common and unique microorganisms present in the two groups. At the species level, 341, 61, 285, and 13 common bacterial, viral, eukaryotic, and archaean species, respectively, were found. The relative abundance of common microorganisms varied between the two groups. In addition to the common microorganisms, there were 23 species of microorganisms unique to the midgut of *H. qinghaiensis* collected from yaks and 32 species of microorganisms unique to the midgut of *H. qinghaiensis* collected from Tibetan sheep. These results suggest that most of the microbial species in *H. qinghaiensis* females collected from different hosts were the same, but there were also slight differences and unique microorganisms found in each host. These findings are similar to the conclusions of Xu et al. [24], who showed that the intestinal microbial diversity of *R. microplus* was influenced by the different hosts analyzed (cattle and goats).

The sources of tick microbial communities are diverse and complex, with specific sources including (1) micro-habitats at the surface of vertebrate hosts, which provide a complex source of organisms for ticks to potentially acquire [25]; (2) exchange through co-feeding as well as from the external environment (considering that ticks spend approximately 90% of their life off the host) [26]; and (3) transmission of microbiomes to the next generation via vertical transmission (bacterial genera that can be vertically transmitted include *Coxiella*-like endosymbiont, *Francisella*-like endosymbiont, *Midichloria*, *Wolbachia*, and *Arsenophonus*) [27]. This makes it likely that factors in tick microbiome variation include species, life stage, sex, degree of engorgement, and geographical location [28–30]. In addition, some studies have demonstrated that the tick microbiome is influenced by both the individual and species identity of the blood meal host [31, 32].

The surface micro-habitat of the vertebrate host, one of the important sources of the tick microbiota, may be affected by host genotype, health status, or host living environments, and thus these factors will indirectly influence the tick microbial community [33]. The hosts of *H. qinghaiensis* ticks collected in this study were yaks and Tibetan sheep. Yaks are distributed in the alpine area above 3000 m above sea level in and around the Qinghai–Tibet Plateau of China. They are highly adaptable to the harsh environments of high altitude, low oxygen, cold climate, and the short growing period of pasture grasses. Similarly, the Tibetan sheep are distributed in the high mountain valley area of 1800–4000 m, and can also adapt to the environment of high altitude, low oxygen, and cold climate. Lazikou Town, Diebu County, Gannan Tibetan Autonomous Prefecture, Gansu Province, China, where the samples of *H. qinghaiensis* were collected, is located in the high mountain valley of the middle reaches of the Bailong River, with average elevation of 2950 m. The average annual temperature is 9 °C; in April, when the ticks were collected, the average maximum and minimum temperatures were 17 °C and −1 °C, respectively. Due to the limited number of samples in this study, more in-depth research is needed to confirm whether the environment can affect the microorganisms in the midgut of *H. qinghaiensis* females collected from yaks and Tibetan sheep. However, it has been confirmed that the dissemination of ticks has begun to expand to higher elevations with the effects of deforestation, increased urbanization, warmer winters, and longer transitional autumn and spring seasons [34, 35]. With this expansion, ticks have increasing access to different microbial communities outside the microbial consortia encountered in traditional geographical regions [26].

Ticks are obligate hematophagous arthropods that rely primarily on host blood for growth and development. This lifestyle is unique, as hematophagous insects feed on blood, a diet that is rich in protein but deficient in essential B vitamins and co-factors, and relatively poor in other nutrients such as lipids [36]. Due to the nutritional limitations of blood meals, hematophagous insects have evolved a suitable way of digesting and utilizing the blood meal. Numerous studies have shown that some of the beneficial symbionts in the tick microbiome have evolved intimate interactions that play important roles in digesting blood meals, providing essential B vitamins and co-factors, and vital nutrients [37–39]. Hematophagy aids in increasing the relative abundance of these beneficial symbionts [40]. In this study, KEGG pathway analysis revealed the functional genes of the microbiome of *H. qinghaiensis* involved in different metabolic pathways including carbohydrates, lipids, amino acids, glycans, nucleotides, energies, and vitamins and co-factors, with a higher number of genes associated with carbohydrate and lipid metabolism. This is similar to Obregón's findings [41] and also suggests that the contribution of tick midgut microbiota can go beyond B vitamin supplementation.

Lipids are a diverse group of molecules with variable structures and multiple metabolic and cellular functions. In insects, lipids from the meal are usually digested in the midgut lumen and absorbed by the midgut epithelium, where they are typically stored in the fat body as lipid droplet-associated triacylglycerols and play an important role in the process of oogenesis [42]. Due to the low lipid content of the host blood, reliance on digested lipids from blood meal cannot satisfy the lipid requirements of hematophagous insects during oogenesis [36]. In the present study, we found that the number of functional genes of the microbiome of *H. qinghaiensis* enriched in lipid metabolism was higher, which will probably provide the required lipids for oogenesis after the ticks are saturated with host blood. At the same time, it has been shown that insects may synthesize lipids de novo using carbohydrates and amino acid substrates, which seems to largely contribute to reproduction [36, 42]. The results of this study showed that the functional genes of the *H. qinghaiensis* microbiome were heavily enriched in carbohydrate metabolism, which would potentially provide a large amount of raw material for de novo lipid synthesis. In addition, several functional genes of the microbiome of *H. qinghaiensis* found in this study have been linked to human diseases, while some microbial species were associated with infectious diseases, further confirming that ticks are biological vectors for the transmission of human and animal diseases [43].

In this study, the relative abundance of *Anaplasma* in Hq. C and Hq. S was 4.5% and 4.3%, respectively, and this was the dominant genus in both groups; the species was identified as *A. phagocytophilum*, which is a tick-borne, specialized intracellular parasitic pathogen that causes human granulocytic anaplasmosis (HGA) [44]. The main symptom of HGA in humans is fever, especially a persistent high fever that is often accompanied by respiratory diseases such as interstitial pneumonia and pulmonary edema [45]. The main symptom of HGA in animals is toxic myocarditis accompanied by liver damage such as mild hepatitis and gastrointestinal damage such as gastrointestinal inflammation [46]. *Anaplasma phagocytophilum* has become an important tick-borne pathogen endangering public health in the United States, Europe, and Asia [47]. *Anaplasma phagocytophilum* has been detected in *R. microplus* [9], *H. longicornis* [8], *Ixodes ricinus* [48], *Ixodes scapularis* [49], and *Dermacentor reticulatus* [50]. In this study, *A. phagocytophilum* was detected in the midgut of *H. qinghaiensis* from different hosts, indicating that this pathogen could be stably colonized in the midgut of *H. qinghaiensis*. Whether there is a risk of transmission remains to be further studied, but it would be prudent to strengthen the prevention and control of *H. qinghaiensis*.

In the present study, *Ehrlichia* was a common bacterial genus in both groups and had a high relative abundance (Hq. C 2.1% and Hq. S 1.9%). *Ehrlichia* is an intracellular gram-negative tick-borne α Proteus [51]. *Ehrlichia chaffeensis*, *E. canis*, and *E. ewingii* in the genus *Ehrlichia* are important pathogens that can be transmitted by ticks and infect dogs and humans, causing febrile diseases such as fever, lethargy, myalgia, and leukopenia [52, 53]. In this study, the genus *Ehrlichia* in both groups was identified as *E. minasensis*, which is a recently discovered pathogen that is closely related to *E. canis* [54]. *Ehrlichia minasensis* has been detected in tick species such as *Hyalomma marginatum* from France [55], *Hyalomma anatolicum* from Pakistan [56], *R. microplus* from Brazil [57], and *Rhipicephalus appendiculatus* from South Africa [58]. In China, Li et al. [59] found the first known naturally occurring *E. minasensis* in *Haemaphysalis hystricis* in Hainan Province. Cao et al. [8] also detected *E. minasensis* in *H. longicornis* in Shanxi Province. The present study found *E. minasensis* in the midgut of *H. qinghaiensis* females collected from different hosts, indicating that *E. minasensis* can colonize the midgut of *H. qinghaiensis*.

*Pseudomonas aeruginosa* is an aerobic and facultative anaerobic Gram-negative bacillus [60], one of the common pathogenic bacteria causing respiratory diseases [61]. It was first isolated from wound pus by Gersard in 1882 [62]. *Pseudomonas aeruginosa* has a wide range of hosts, including aquatic and terrestrial plants, animals, and humans [62]. It can cause various acute and chronic infections in poultry, livestock, and pets, and can develop into diseases such as fatal diarrhea, bacteremia, and sepsis [61]. This pathogen seriously endangers animal health and causes significant economic losses to the animal production and breeding industries [63]. Meanwhile, *P. aeruginosa* and its metabolically produced toxins and waste products can be transmitted to the human body through direct contact or meat consumption, posing a risk to human health [63]. *Pseudomonas aeruginosa* has been detected in *H. flava*, *R. microplus*, and *Dermacentor variabilis* [64–66]. In this study, *P. aeruginosa* was detected in the midgut of fully engorged *H. qinghaiensis* females collected from yaks and Tibetan sheep. The relative abundance of *P. aeruginosa* in the midgut of *H. qinghaiensis* females collected from different hosts was high, and the difference in relative abundance was relatively small between the two groups. This indicates that *H. qinghaiensis* collected from different hosts carried a certain abundance of *P. aeruginosa*.

*Staphylococcus aureus* is a Gram-positive pathogenic bacterium. Both humans and animals are the main carriers of *S. aureus* [67, 68]. This bacterium is an important veterinary pathogen that can cause severe invasive infections [69] such as pneumonia, pericarditis, and even systemic infectious diseases such as sepsis and septicemia as well as suppurative inflammation of a disseminated nature such as canker sores, otitis media, and osteomyelitis [70]. Infection with *S. aureus* can lead to diseases such as arthritis, osteomyelitis, mastitis, and lobar pneumonia in domestic animals [70]. *Staphylococcus aureus* has been detected in *R. microplus* [6], *Argas persicus* [71], and house dust mites [72]. In the present study, *S. aureus* was detected in the midgut of *H. qinghaiensis* females collected from yaks and Tibetan sheep, and the relative abundance difference between the two groups was relatively small (Hq. C 1.1% and Hq. S 1.2%). This indicates that *S. aureus* could stably colonize the midgut of *H. qinghaiensis*, and the relative abundance of *S. aureus* in the *H. qinghaiensis* midgut did not vary between the different hosts. Meanwhile, although *S. aureus* is part of the normal microflora of human and animal skins, it is still at high prevalence in ticks and may cause host diseases through transmission via blood feeding.

Orf virus is a species of the genus *Parapoxvirus*, which is a pathogen of infectious pustular diseases that cause economic losses in livestock production [73]. This virus primarily infects sheep and goats, but infections have also been reported in various ruminants and other mammals, and this virus can be transmitted to humans through direct or indirect contact with infected animals [73]. Orf virus often causes pustular dermatitis in humans, sheep, and goats [74]. Erythema, pimples, vesicles, pustules, and scabs appear when the damaged skin is infected by the orf virus, but the infection is confined to the epidermis, and there is no sign of systematic transmission [75]. In addition to direct transmission, the orf virus can be transmitted through the bites of ticks [76]. The orf virus has been detected in *R. microplus* [9]. In this study, the orf virus was detected in the midgut of *H. qinghaiensis* females collected from yaks and Tibetan sheep, and its relative abundance was higher than that of other viruses in the two groups. Meanwhile, the relative abundance of the orf virus in the midgut of *H. qinghaiensis* females collected from yaks (0.1%) and Tibetan sheep (0.08%) was not significantly different. These results indicate that the orf virus can colonize the midgut of *H. qinghaiensis*, and *H. qinghaiensis* collected from different hosts carried a certain abundance of orf virus.

Ascoviruses are a group of large DNA viruses that infect Lepidoptera insects and are transmitted by endoparasitic wasps. Wang et al. [77] found Ascoviruses in *Dasineura jujubifolia*, suggesting that Ascoviruses may be distributed in a much wider range of insects than previously known. Ascoviruses were only detected in the sample of *H. qinghaiensis* collected from Tibetan sheep, not in the sample from yaks, which may be due to differences in host genotypes, health status, or host living environments [33]. In addition, *Clostridium paraputrificum* and *Hypoxylon* sp. CI-4A were only found in the sample of *H. qinghaiensis* collected from yaks; *Vibrio ouci*, *Hyphodiscus hymeniophilus*, and bat gammaretrovirus were only detected in the sample of *H. qinghaiensis* collected from Tibetan sheep. This suggests that there are slight differences in the midgut microbial community composition of the *H. qinghaiensis* collected from different hosts.

In this study, we collected 50 ticks from both yak and Tibetan sheep and then pooled five fully engorged female ticks together. However, from a statistical perspective, these pooled ticks essentially represent a single observation. Given this limitation, it is not feasible to conduct a robust comparison regarding the prevalence of specific communities between the two groups and to analyze the differences in gene function in depth. Hence, this study mainly investigated the microbial species present in the midgut microbial community of fully engorged *H. qinghaiensis* females collected from yaks and Tibetan sheep. In future research, we will increase the sample size, and ticks in different groups will be analyzed as individual samples to clarify whether the microbial community of the same tick species can be affected by different hosts. Although this study has some limitations, the data can still provide some reference for understanding the midgut microbial composition of *H. qinghaiensis* and predicting tick-borne diseases.

## Conclusions

In this study, we analyzed the midgut microbial composition of fully engorged *H. qinghaiensis* females collected from yaks and Tibetan sheep using metagenomic sequencing technology. Overall, 57 phyla, 483 genera, and 755 species were identified in the two groups. The microbial composition of the two groups included not only a large number of bacteria but also eukaryotes, viruses, and a few archaea. Most of the microbial species were the same in the two groups, but there were slight differences. We also found that the functional genes of the microbiome of *H. qinghaiensis* annotated to carbohydrate metabolism and lipid metabolism were more abundant than other functional genes in the metabolic pathway. The microbiome functional genes associated with infectious diseases were more abundant in the human disease pathway. These findings add to the physiological information for *H. qinghaiensis* and whole ticks, and the results provide a foundation for the prevention and control of ticks and tick-borne diseases.

### Supplementary Information

Additional file 1: Table S1. Relative abundance of other than the top 20 common phyla in the two groups of *Haemaphysalis qinghaiensis*.

Additional file 2: Table S2. Relative abundance of other than the top 20 common genera in the two groups of *Haemaphysalis qinghaiensis*.

Additional file 3: Table S3. Relative abundance of other than the top 25 common bacterial species in the two groups of *Haemaphysalis qinghaiensis*.

Additional file 4: Table S4. Relative abundance of other than the top 10 common viral species in the two groups of *Haemaphysalis qinghaiensis*.

Additional file 5: Table S5. Relative abundance of the common eukaryotic species in the two groups of *Haemaphysalis qinghaiensis*.

Additional file 6: Table S6. Relative abundance of the common archaean species in the two groups of *Haemaphysalis qinghaiensis*.

## Data Availability

The raw tags of the midgut of *Haemaphysalis qinghaiensis* females collected from yaks and Tibetan sheep have been deposited in the Sequence Read Archive (SRA) of the NCBI under BioProject accession numbers PRJNA1043882 and PRJNA1037618, respectively. The individual run files received accession numbers SAMN38357070, SAMN38357071, SAMN38357072, SAMN38341130, SAMN38341131, and SAMN38341132.

## Abbreviations

KEGG: Kyoto Encyclopedia of Genes and Genomes
PCR: Polymerase chain reaction
PCR-DGGE: Polymerase chain reaction–denaturing gradient gel electrophoresis
CTAB: Hexadecyltrimethylammonium bromide
HGA: Human granulocytic anaplasmosis
