# Supplementary material for: Metagenome reveals the midgut microbial community of Haemaphysalis qinghaiensis ticks collected from yaks and Tibetan sheep
Source: Parasit Vectors. 2024 Aug 31;17:370. doi: 10.1186/s13071-024-06442-y (PMC11366167; doi:10.1186/s13071-024-06442-y)
Supplement: Supplementary file 1 — Additional file 1: Table S1. Relative abundance of other than the top 20 common phyla in the two groups of Haemaphysalis qinghaiensis. [file 13071_2024_6442_MOESM1_ESM.docx]

**Additional File 1: Table S1.** Relative abundance of other than the top 20 common phyla in the two groups of *Haemaphysalis qinghaiensis*.

| Kingdom | Phylum | Abundance (%) | |
| --- | --- | --- | --- |
|  |  | Hq. C | Hq. S |
| Archaea | Candidatus Heimdallarchaeota | 0.00004170 | 0.00003560 |
|  | Candidatus Nezhaarchaeota | 0.00000212 | 0.00000183 |
|  | Candidatus Thermoplasmatota | 0.00001010 | 0.00001280 |
|  | Crenarchaeota | 0.00000099 | 0.00000047 |
|  | Euryarchaeota | 0.00001580 | 0.00001220 |
|  | Thaumarchaeota | 0.00000469 | 0.00000324 |
| Bacteria | Acidobacteria | 0.00001540 | 0.00001430 |
|  | Armatimonadetes | 0.00000122 | 0.00000347 |
|  | Caldiserica | 0.00000042 | 0.00000110 |
|  | Candidatus Blackallbacteria | 0.00000768 | 0.00000604 |
|  | Candidatus Dadabacteria | 0.00000040 | 0.00000096 |
|  | Candidatus Eremiobacteraeota | 0.00000107 | 0.00000203 |
|  | Candidatus Marinimicrobia | 0.00000120 | 0.00000179 |
|  | Candidatus Pacebacteria | 0.00000093 | 0.00000124 |
|  | Candidatus Parcubacteria | 0.00000140 | 0.00000179 |
|  | Candidatus Rokubacteria | 0.00000055 | 0.00000130 |
|  | Chlamydiae | 0.00002630 | 0.00002010 |
|  | Chlorobi | 0.00000100 | 0.00000116 |
|  | Chloroflexi | 0.00003740 | 0.00003440 |
|  | Deinococcus-Thermus | 0.00000042 | 0.00000201 |
|  | Fusobacteria | 0.00000173 | 0.00000288 |
|  | Gemmatimonadetes | 0.00000304 | 0.00000160 |
|  | Nitrospirae | 0.00000070 | 0.00000110 |
|  | Planctomycetes | 0.00006460 | 0.00006200 |
|  | Rhodothermaeota | 0.00000114 | 0.00000108 |
|  | Verrucomicrobia | 0.00002930 | 0.00002790 |
| Eukaryota | Blastocladiomycota | 0.00002270 | 0.00003030 |
|  | Cryptomycota | 0.00002730 | 0.00003320 |
|  | Olpidiomycota | 0.00000572 | 0.00000736 |
| Viruses | Cossaviricota | 0.00001490 | 0.00001250 |
|  | Duplornaviricota | 0.00001210 | 0.00001620 |
|  | Peploviricota | 0.00001890 | 0.00001530 |
|  | Uroviricota | 0.00000684 | 0.00000773 |
