# Supplementary material for: Metagenome reveals the midgut microbial community of Haemaphysalis qinghaiensis ticks collected from yaks and Tibetan sheep
Source: Parasit Vectors. 2024 Aug 31;17:370. doi: 10.1186/s13071-024-06442-y (PMC11366167; doi:10.1186/s13071-024-06442-y)
Supplement: Supplementary file 2 — Additional file 2: Table S2. Relative abundance of other than the top 20 common genera in the two groups of Haemaphysalis qinghaiensis. [file 13071_2024_6442_MOESM2_ESM.docx]

**Additional File 2: Table S2.** Relative abundance of other than the top 20 common genera in the two groups of *Haemaphysalis qinghaiensis*.

| Kingdom | Genus | Abundance (%) | |
| --- | --- | --- | --- |
|  |  | Hq. C | Hq. S |
| Archaea | *Halomicroarcula* | 0.00000245 | 0.00000146 |
|  | *Halorubrum* | 0.00000186 | 0.00000127 |
|  | *Natronococcus* | 0.00000065 | 0.00000062 |
|  | *Nitrosopumilus* | 0.00000113 | 0.00000027 |
|  | *Thermococcus* | 0.00000283 | 0.00000246 |
|  | *Thermoproteus* | 0.00000099 | 0.00000047 |
| Bacteria | *Acidobacterium* | 0.00000024 | 0.00000139 |
|  | *Acidovorax* | 0.00000267 | 0.00000085 |
|  | *Actinoplanes* | 0.00000197 | 0.00000063 |
|  | *Actinopolymorpha* | 0.00000079 | 0.00000477 |
|  | *Aequorivita* | 0.00000098 | 0.00000246 |
|  | *Aeromicrobium* | 0.00000851 | 0.00000678 |
|  | *Aeromonas* | 0.00004950 | 0.00004500 |
|  | *Alcanivorax* | 0.00001460 | 0.00001430 |
|  | *Algoriphagus* | 0.00000083 | 0.00000118 |
|  | *Alicyclobacillus* | 0.00000342 | 0.00000341 |
|  | *Aliidiomarina* | 0.00000114 | 0.00000027 |
|  | *Aliidongia* | 0.00040239 | 0.00042700 |
|  | *Alkalispirillum* | 0.00000140 | 0.00000222 |
|  | *Alkalispirochaeta* | 0.00000230 | 0.00000036 |
|  | *Allokutzneria* | 0.00000048 | 0.00000092 |
|  | *Alteriqipengyuania* | 0.00000044 | 0.00000166 |
|  | *Amycolatopsis* | 0.00000297 | 0.00000063 |
|  | *Anabaena* | 0.00000090 | 0.00000107 |
|  | *Anaerosalibacter* | 0.00000103 | 0.00000033 |
|  | *Aquifex* | 0.00063479 | 0.00058146 |
|  | *Arsenophonus* | 0.00000318 | 0.00000771 |
|  | *Aureimonas* | 0.00003330 | 0.00003510 |
|  | *Bacillus* | 0.00092167 | 0.00061991 |
|  | *Borreliella* | 0.00001650 | 0.00002250 |
|  | *Bowmanella* | 0.00000404 | 0.00000100 |
|  | *Brachybacterium* | 0.00000109 | 0.00000207 |
|  | *Brachyspira* | 0.00049746 | 0.00037089 |
|  | *Bradyrhizobium* | 0.00000552 | 0.00000792 |
|  | *Brevibacillus* | 0.00000165 | 0.00000148 |
|  | *Burkholderia* | 0.00000568 | 0.00000853 |
|  | *Butyricimonas* | 0.00000194 | 0.00000456 |
|  | *Campylobacter* | 0.00018949 | 0.00025080 |
|  | *Candidatus* Hodgkinia | 0.00057309 | 0.00033850 |
|  | *Candidatus* Magnetomorum | 0.00000165 | 0.00000067 |
|  | *Candidatus* Nephrothrix | 0.00011858 | 0.00009370 |
|  | *Candidatus* Regiella | 0.00004220 | 0.00004830 |
|  | *Capsulimonas* | 0.00000038 | 0.00000164 |
|  | *Carnobacterium* | 0.00012479 | 0.00010722 |
|  | *Catellatospora* | 0.00000397 | 0.00000200 |
|  | *Cecembia* | 0.00000154 | 0.00000037 |
|  | *Cellulomonas* | 0.00000209 | 0.00000240 |
|  | *Chitinophaga* | 0.00001010 | 0.00001280 |
|  | *Chlamydia* | 0.00002540 | 0.00001830 |
|  | *Chlorobium* | 0.00000100 | 0.00000116 |
|  | *Christensenella* | 0.00000118 | 0.00000063 |
|  | *Chryseobacterium* | 0.00000510 | 0.00000309 |
|  | *Citrobacter* | 0.00013982 | 0.00014238 |
|  | *Clostridioides* | 0.00009070 | 0.00033601 |
|  | *Cohnella* | 0.00000304 | 0.00000076 |
|  | *Comamonas* | 0.00000102 | 0.00000169 |
|  | *Commensalibacter* | 0.00000026 | 0.00000176 |
|  | *Corynebacterium* | 0.00007330 | 0.00004350 |
|  | *Crocosphaera* | 0.00003270 | 0.00003070 |
|  | *Cupriavidus* | 0.00000060 | 0.00000128 |
|  | *Curtobacterium* | 0.00001280 | 0.00000868 |
|  | *Cyanothece* | 0.00000609 | 0.00000819 |
|  | *Cyclobacterium* | 0.00000348 | 0.00000028 |
|  | *Cycloclasticus* | 0.00007660 | 0.00007220 |
|  | *Derxia* | 0.00000262 | 0.00000116 |
|  | *Desulfobacula* | 0.00000051 | 0.00000242 |
|  | *Desulfocapsa* | 0.00024692 | 0.00025452 |
|  | *Desulfosediminicola* | 0.00000235 | 0.00000196 |
|  | *Devosia* | 0.00000032 | 0.00000090 |
|  | *Dongshaea* | 0.00000037 | 0.00000094 |
|  | *Eggerthia* | 0.00010474 | 0.00010398 |
|  | *Elioraea* | 0.00000361 | 0.00000420 |
|  | *Endozoicomonas* | 0.00000429 | 0.00000191 |
|  | *Enhygromyxa* | 0.00000178 | 0.00000153 |
|  | *Enterobacter* | 0.00067848 | 0.00058024 |
|  | *Enterovibrio* | 0.00001360 | 0.00001270 |
|  | *Epulopiscium* | 0.00017829 | 0.00016697 |
|  | *Erwinia* | 0.00000977 | 0.00001220 |
|  | *Escherichia* | 0.00091534 | 0.00095484 |
|  | *Flammeovirga* | 0.00000437 | 0.00000161 |
|  | *Fontimonas* | 0.00000165 | 0.00000079 |
|  | *Francisella* | 0.00000241 | 0.00000535 |
|  | *Fusobacterium* | 0.00000173 | 0.00000288 |
|  | *Gloeocapsa* | 0.00000193 | 0.00000307 |
|  | *Gloeothece* | 0.00000942 | 0.00001120 |
|  | *Glycocaulis* | 0.00000436 | 0.00000439 |
|  | *Haemophilus* | 0.00000083 | 0.00000026 |
|  | *Hahella* | 0.00000452 | 0.00001020 |
|  | *Haliangium* | 0.00000202 | 0.00000192 |
|  | *Haloferula* | 0.00000033 | 0.00000125 |
|  | *Halomonas* | 0.00000122 | 0.00000261 |
|  | *Halopseudomonas* | 0.00000550 | 0.00000220 |
|  | *Halosaccharopolyspora* | 0.00000235 | 0.00000149 |
|  | *Helicobacter* | 0.00000102 | 0.00000145 |
|  | *Herbaspirillum* | 0.00000271 | 0.00000258 |
|  | *Hydrocarboniphaga* | 0.00000072 | 0.00000103 |
|  | *Hyphomonas* | 0.00000159 | 0.00000038 |
|  | *Jannaschia* | 0.00000206 | 0.00000168 |
|  | *Ketobacter* | 0.00000265 | 0.00000268 |
|  | *Kitasatospora* | 0.00000229 | 0.00000194 |
|  | *Ktedonobacter* | 0.00000250 | 0.00000232 |
|  | *Labedella* | 0.00003760 | 0.00003460 |
|  | *Lacticaseibacillus* | 0.00000332 | 0.00000158 |
|  | *Lactiplantibacillus* | 0.00000020 | 0.00000111 |
|  | *Legionella* | 0.00001880 | 0.00001500 |
|  | *Leifsonia* | 0.00000220 | 0.00000197 |
|  | *Lewinella* | 0.00000031 | 0.00000147 |
|  | *Lujinxingia* | 0.00000165 | 0.00000234 |
|  | *Lyngbya* | 0.00000171 | 0.00000209 |
|  | *Macrococcus* | 0.00000434 | 0.00000174 |
|  | *Marinagarivorans* | 0.00000055 | 0.00000235 |
|  | *Marinifilum* | 0.00005780 | 0.00005500 |
|  | *Marinococcus* | 0.00000056 | 0.00000081 |
|  | *Massilia* | 0.00000083 | 0.00000237 |
|  | *Meiothermus* | 0.00000042 | 0.00000201 |
|  | *Membranicola* | 0.00000192 | 0.00000209 |
|  | *Mesorhizobium* | 0.00000332 | 0.00000045 |
|  | *Methylotenera* | 0.00000092 | 0.00000175 |
|  | *Microbacterium* | 0.00007320 | 0.00007380 |
|  | *Microcystis* | 0.00001200 | 0.00000854 |
|  | *Micromonospora* | 0.00006360 | 0.00005400 |
|  | *Microvirga* | 0.00000021 | 0.00000129 |
|  | *Minicystis* | 0.00000173 | 0.00000156 |
|  | *Moorena* | 0.00000819 | 0.00000868 |
|  | *Morganella* | 0.00004400 | 0.00003380 |
|  | *Mycoavidus* | 0.00000166 | 0.00000316 |
|  | *Mycobacterium* | 0.00066751 | 0.00059469 |
|  | *Mycolicibacterium* | 0.00015336 | 0.00015272 |
|  | *Nannocystis* | 0.00000701 | 0.00000580 |
|  | *Neisseria* | 0.00002370 | 0.00002750 |
|  | *Neobacillus* | 0.00000213 | 0.00000015 |
|  | *Nitratireductor* | 0.00011465 | 0.00011905 |
|  | *Nitrosococcus* | 0.00000018 | 0.00000083 |
|  | *Nocardia* | 0.00007640 | 0.00011484 |
|  | *Nocardiopsis* | 0.00000071 | 0.00000023 |
|  | *Nodularia* | 0.00000125 | 0.00000053 |
|  | *Nonomuraea* | 0.00000029 | 0.00000149 |
|  | *Novosphingobium* | 0.00000079 | 0.00000165 |
|  | *Oceanibaculum* | 0.00000086 | 0.00000115 |
|  | *Oceanicoccus* | 0.00000327 | 0.00000155 |
|  | *Oceaniferula* | 0.00000068 | 0.00000024 |
|  | *Oceanimonas* | 0.00000259 | 0.00000141 |
|  | *Oceanospirillum* | 0.00008480 | 0.00007680 |
|  | *Oxynema* | 0.00000230 | 0.00000123 |
|  | *Paenibacillus* | 0.00000625 | 0.00000538 |
|  | *Panacagrimonas* | 0.00000047 | 0.00000296 |
|  | *Pantoea* | 0.00085384 | 0.00081414 |
|  | *Paracoccus* | 0.00000066 | 0.00000249 |
|  | *Pararhizobium* | 0.00000160 | 0.00000177 |
|  | *Pararhodobacter* | 0.00000180 | 0.00000062 |
|  | *Pasteurella* | 0.00000216 | 0.00000176 |
|  | *Pedobacter* | 0.00000378 | 0.00000309 |
|  | *Phenylobacterium* | 0.00000078 | 0.00000130 |
|  | *Planctomyces* | 0.00000257 | 0.00000244 |
|  | *Polaribacter* | 0.00001280 | 0.00001370 |
|  | *Polyangium* | 0.00000087 | 0.00000107 |
|  | *Posidoniimonas* | 0.00000091 | 0.00000086 |
|  | *Prosthecobacter* | 0.00000150 | 0.00000057 |
|  | *Pseudoalteromonas* | 0.00000576 | 0.00000397 |
|  | *Pseudobacteriovorax* | 0.00000136 | 0.00000129 |
|  | *Pyxidicoccus* | 0.00000089 | 0.00000135 |
|  | *Raoultella* | 0.00000183 | 0.00000104 |
|  | *Reyranella* | 0.00000089 | 0.00000099 |
|  | *Rhabdochromatium* | 0.00003150 | 0.00002290 |
|  | *Rhizobium* | 0.00000218 | 0.00000449 |
|  | *Rhodococcus* | 0.00005150 | 0.00004280 |
|  | *Rhodothermus* | 0.00000172 | 0.00000278 |
|  | *Richelia* | 0.00000236 | 0.00000096 |
|  | *Riemerella* | 0.00000072 | 0.00000102 |
|  | *Rippkaea* | 0.00000078 | 0.00000093 |
|  | *Rubritalea* | 0.00000128 | 0.00000081 |
|  | *Rubrivivax* | 0.00000851 | 0.00000830 |
|  | *Saccharibacillus* | 0.00000021 | 0.00000182 |
|  | *Saccharopolyspora* | 0.00000424 | 0.00000426 |
|  | *Salinispora* | 0.00000335 | 0.00000204 |
|  | *Salinivenus* | 0.00000114 | 0.00000108 |
|  | *Salmonella* | 0.00015219 | 0.00012397 |
|  | *Schaalia* | 0.00002260 | 0.00002050 |
|  | *Scopulibacillus* | 0.00000235 | 0.00000402 |
|  | *Scytonema* | 0.00000028 | 0.00000132 |
|  | *Segetibacter* | 0.00000971 | 0.00000986 |
|  | *Soehngenia* | 0.00000125 | 0.00000134 |
|  | *Sorangium* | 0.00000671 | 0.00000398 |
|  | *Sphingobium* | 0.00000614 | 0.00000546 |
|  | *Sphingomonas* | 0.00000145 | 0.00000184 |
|  | *Spirulina* | 0.00000078 | 0.00000018 |
|  | *Sporolactobacillus* | 0.00000020 | 0.00000133 |
|  | *Streptomyces* | 0.00000905 | 0.00001310 |
|  | *Symmachiella* | 0.00000113 | 0.00000036 |
|  | *Tamlana* | 0.00000282 | 0.00000810 |
|  | *Terracidiphilus* | 0.00000032 | 0.00000120 |
|  | *Tessaracoccus* | 0.00000114 | 0.00000072 |
|  | *Thermosynechococcus* | 0.00000202 | 0.00000048 |
|  | *Thiohalocapsa* | 0.00000162 | 0.00000176 |
|  | *Thiothrix* | 0.00008080 | 0.00008250 |
|  | *Tissierella* | 0.00000058 | 0.00000165 |
|  | *Treponema* | 0.00000113 | 0.00000089 |
|  | *Trichococcus* | 0.00000124 | 0.00000094 |
|  | *Tsukamurella* | 0.00000178 | 0.00000228 |
|  | *Tuwongella* | 0.00000029 | 0.00000111 |
|  | *Variovorax* | 0.00000062 | 0.00000107 |
|  | *Varunaivibrio* | 0.00000087 | 0.00000221 |
|  | *Vibrio* | 0.00005360 | 0.00005440 |
|  | *Virgibacillus* | 0.00001360 | 0.00001370 |
|  | *Vitiosangium* | 0.00000111 | 0.00000247 |
|  | *Wolbachia* | 0.00059816 | 0.00057637 |
|  | *Xanthomonas* | 0.00025021 | 0.00025877 |
|  | *Xylella* | 0.00000207 | 0.00000083 |
|  | *Yersinia* | 0.00000178 | 0.00000127 |
|  | *Zooshikella* | 0.00000264 | 0.00000276 |
|  | *Zymomonas* | 0.00000244 | 0.00000103 |
| Eukaryota | *Absidia* | 0.00002100 | 0.00002270 |
|  | *Acaromyces* | 0.00000264 | 0.00000126 |
|  | *Acaulospora* | 0.00011630 | 0.00011258 |
|  | *Actinomortierella* | 0.00001190 | 0.00000908 |
|  | *Agaricus* | 0.00001110 | 0.00000851 |
|  | *Agrocybe* | 0.00001020 | 0.00000457 |
|  | *Akanthomyces* | 0.00006560 | 0.00006920 |
|  | *Allomyces* | 0.00000639 | 0.00000454 |
|  | *Alternaria* | 0.00000291 | 0.00000188 |
|  | *Amanita* | 0.00001170 | 0.00001050 |
|  | *Ambispora* | 0.00010870 | 0.00009320 |
|  | *Amphiamblys* | 0.00000206 | 0.00000112 |
|  | *Anaeromyces* | 0.00001350 | 0.00000840 |
|  | *Anncaliia* | 0.00001020 | 0.00000722 |
|  | *Antrodiella* | 0.00000194 | 0.00000053 |
|  | *Apophysomyces* | 0.00002420 | 0.00002180 |
|  | *Ascobolus* | 0.00000299 | 0.00000328 |
|  | *Ascosphaera* | 0.00000339 | 0.00000288 |
|  | *Aspergillus* | 0.00001340 | 0.00001550 |
|  | *Basidiobolus* | 0.00005610 | 0.00005850 |
|  | *Batrachochytrium* | 0.00002500 | 0.00002330 |
|  | *Bifiguratus* | 0.00000697 | 0.00000749 |
|  | *Bipolaris* | 0.00000123 | 0.00000047 |
|  | *Blyttiomyces* | 0.00000549 | 0.00000399 |
|  | *Boletus* | 0.00000028 | 0.00000122 |
|  | *Cadophora* | 0.00000099 | 0.00000158 |
|  | *Calocera* | 0.00001820 | 0.00001160 |
|  | *Candida* | 0.00000154 | 0.00000173 |
|  | *Candolleomyces* | 0.00000059 | 0.00000254 |
|  | *Cantharellus* | 0.00000106 | 0.00000189 |
|  | *Capronia* | 0.00000080 | 0.00000044 |
|  | *Catenaria* | 0.00001520 | 0.00002490 |
|  | *Caulochytrium* | 0.00000493 | 0.00000459 |
|  | *Ceraceosorus* | 0.00000602 | 0.00000341 |
|  | *Ceratobasidium* | 0.00015488 | 0.00013083 |
|  | *Cercospora* | 0.00001800 | 0.00001390 |
|  | *Cetraspora* | 0.00002770 | 0.00002500 |
|  | *Chaetomium* | 0.00000425 | 0.00000795 |
|  | *Chalara* | 0.00000027 | 0.00000182 |
|  | *Chlorociboria* | 0.00000276 | 0.00000117 |
|  | *Choanephora* | 0.00003320 | 0.00003650 |
|  | *Choiromyces* | 0.00000306 | 0.00000367 |
|  | *Chytriomyces* | 0.00000643 | 0.00001050 |
|  | *Claroideoglomus* | 0.00031411 | 0.00029155 |
|  | *Claviceps* | 0.00000140 | 0.00000346 |
|  | *Clavispora* | 0.00000108 | 0.00000123 |
|  | *Coemansia* | 0.00000725 | 0.00000682 |
|  | *Coleophoma* | 0.00000141 | 0.00000027 |
|  | *Colletotrichum* | 0.00000422 | 0.00000451 |
|  | *Conidiobolus* | 0.00000540 | 0.00000732 |
|  | *Coniochaeta* | 0.00000270 | 0.00000085 |
|  | *Coprinopsis* | 0.00001620 | 0.00002230 |
|  | *Cronartium* | 0.00000336 | 0.00000269 |
|  | *Cryptococcus* | 0.00001060 | 0.00000910 |
|  | *Cucumispora* | 0.00054522 | 0.00045158 |
|  | *Cylindrobasidium* | 0.00000196 | 0.00000196 |
|  | *Daedalea* | 0.00000107 | 0.00000058 |
|  | *Dentipellis* | 0.00000061 | 0.00000138 |
|  | *Dentiscutata* | 0.00010570 | 0.00010362 |
|  | *Dictyocoela* | 0.00055152 | 0.00054522 |
|  | *Dimargaris* | 0.00001140 | 0.00000574 |
|  | *Dissophora* | 0.00000091 | 0.00000159 |
|  | *Diutina* | 0.00000040 | 0.00000096 |
|  | *Diversispora* | 0.00006350 | 0.00006060 |
|  | *Endogone* | 0.00001350 | 0.00001390 |
|  | *Enterospora* | 0.00013031 | 0.00012237 |
|  | *Entomophthora* | 0.00000539 | 0.00000555 |
|  | *Entomortierella* | 0.00001210 | 0.00001390 |
|  | *Erysiphe* | 0.00006210 | 0.00006490 |
|  | *Escovopsis* | 0.00000557 | 0.00000310 |
|  | *Exidia* | 0.00000063 | 0.00000100 |
|  | *Exophiala* | 0.00000050 | 0.00000079 |
|  | *Fistulina* | 0.00000123 | 0.00000047 |
|  | *Friedmanniomyces* | 0.00000193 | 0.00000071 |
|  | *Fulvia* | 0.00001220 | 0.00000867 |
|  | *Funneliformis* | 0.00001220 | 0.00001090 |
|  | *Furculomyces* | 0.00000066 | 0.00000079 |
|  | *Fusarium* | 0.00005360 | 0.00004030 |
|  | *Gaeumannomyces* | 0.00000083 | 0.00000118 |
|  | *Gamsiella* | 0.00000229 | 0.00000188 |
|  | *Ganoderma* | 0.00000253 | 0.00000342 |
|  | *Geosiphon* | 0.00003930 | 0.00003550 |
|  | *Geosmithia* | 0.00000089 | 0.00000021 |
|  | *Geotrichum* | 0.00000266 | 0.00000179 |
|  | *Gigaspora* | 0.00003830 | 0.00003220 |
|  | *Gloeophyllum* | 0.00000223 | 0.00000061 |
|  | *Glomus* | 0.00000266 | 0.00000144 |
|  | *Gonapodya* | 0.00004480 | 0.00003720 |
|  | *Gryganskiella* | 0.00000183 | 0.00000119 |
|  | *Gymnopilus* | 0.00002870 | 0.00002890 |
|  | *Hamiltosporidium* | 0.00002780 | 0.00002600 |
|  | *Haplosporangium* | 0.00000923 | 0.00001060 |
|  | *Hepatospora* | 0.00001170 | 0.00001000 |
|  | *Hericium* | 0.00000277 | 0.00000244 |
|  | *Hermanssonia* | 0.00021024 | 0.00019964 |
|  | *Hesseltinella* | 0.00000315 | 0.00000392 |
|  | *Hypsizygus* | 0.00000085 | 0.00000182 |
|  | *Jimgerdemannia* | 0.00001060 | 0.00001120 |
|  | *Kazachstania* | 0.00000499 | 0.00000788 |
|  | *Kluyveromyces* | 0.00000226 | 0.00000172 |
|  | *Kuraishia* | 0.00000110 | 0.00000023 |
|  | *Kwoniella* | 0.00002450 | 0.00002900 |
|  | *Laccaria* | 0.00000428 | 0.00000298 |
|  | *Laetiporus* | 0.00000124 | 0.00000196 |
|  | *Lasallia* | 0.00000390 | 0.00000346 |
|  | *Lentinula* | 0.00002500 | 0.00002690 |
|  | *Leucosporidium* | 0.00000060 | 0.00000114 |
|  | *Lichtheimia* | 0.00004640 | 0.00003890 |
|  | *Linderina* | 0.00000850 | 0.00000554 |
|  | *Linnemannia* | 0.00000524 | 0.00000463 |
|  | *Lipomyces* | 0.00000320 | 0.00000121 |
|  | *Lobosporangium* | 0.00000313 | 0.00000089 |
|  | *Lophiotrema* | 0.00000192 | 0.00000125 |
|  | *Macrophomina* | 0.00001330 | 0.00001690 |
|  | *Malassezia* | 0.00000242 | 0.00000243 |
|  | *Melanogaster* | 0.00000109 | 0.00000052 |
|  | *Melanopsichium* | 0.00000303 | 0.00000081 |
|  | *Metarhizium* | 0.00003470 | 0.00002710 |
|  | *Microbotryum* | 0.00003460 | 0.00003330 |
|  | *Mixia* | 0.00000046 | 0.00000227 |
|  | *Moesziomyces* | 0.00000118 | 0.00000028 |
|  | *Monilinia* | 0.00000171 | 0.00000102 |
|  | *Monosporascus* | 0.00001530 | 0.00001630 |
|  | *Morchella* | 0.00000997 | 0.00001140 |
|  | *Mortierella* | 0.00002840 | 0.00002960 |
|  | *Mucidula* | 0.00000133 | 0.00000098 |
|  | *Mucor* | 0.00046912 | 0.00037638 |
|  | *Mycena* | 0.00000441 | 0.00000363 |
|  | *Myriangium* | 0.00000037 | 0.00000107 |
|  | *Mytilinidion* | 0.00000729 | 0.00000683 |
|  | *Naganishia* | 0.00000099 | 0.00000095 |
|  | *Nannizzia* | 0.00000478 | 0.00000630 |
|  | *Neocallimastix* | 0.00002090 | 0.00002190 |
|  | *Neolecta* | 0.00000222 | 0.00000192 |
|  | *Nosema* | 0.00050396 | 0.00048796 |
|  | *Ogataea* | 0.00000412 | 0.00000635 |
|  | *Olpidium* | 0.00000572 | 0.00000736 |
|  | *Ophiostoma* | 0.00000311 | 0.00000171 |
|  | *Orpinomyces* | 0.00000118 | 0.00000224 |
|  | *Paraglomus* | 0.00008880 | 0.00009280 |
|  | *Paramicrosporidium* | 0.00000309 | 0.00000434 |
|  | *Parasitella* | 0.00002010 | 0.00001900 |
|  | *Patellaria* | 0.00000195 | 0.00000093 |
|  | *Penicillium* | 0.00008030 | 0.00009170 |
|  | *Phanerochaete* | 0.00000190 | 0.00000119 |
|  | *Phycomyces* | 0.00001250 | 0.00001050 |
|  | *Piptocephalis* | 0.00000167 | 0.00000126 |
|  | *Piromyces* | 0.00001940 | 0.00002870 |
|  | *Pneumocystis* | 0.00000124 | 0.00000231 |
|  | *Podila* | 0.00001450 | 0.00002040 |
|  | *Polyplosphaeria* | 0.00000072 | 0.00000103 |
|  | *Polyporus* | 0.00000143 | 0.00000119 |
|  | *Powellomyces* | 0.00000681 | 0.00001210 |
|  | *Pseudogymnoascus* | 0.00020906 | 0.00016656 |
|  | *Psilocybe* | 0.00000168 | 0.00000032 |
|  | *Pterula* | 0.00000364 | 0.00000133 |
|  | *Puccinia* | 0.00013347 | 0.00012166 |
|  | *Pyronema* | 0.00000097 | 0.00000037 |
|  | *Pyrrhoderma* | 0.00000105 | 0.00000025 |
|  | *Racocetra* | 0.00000126 | 0.00000315 |
|  | *Rhizoclosmatium* | 0.00000756 | 0.00000268 |
|  | *Rhizoctonia* | 0.00003690 | 0.00004620 |
|  | *Rhizopus* | 0.00048581 | 0.00043001 |
|  | *Rhodotorula* | 0.00000568 | 0.00000967 |
|  | *Rozella* | 0.00002420 | 0.00002890 |
|  | *Russula* | 0.00000381 | 0.00000300 |
|  | *Saccharomycodes* | 0.00000092 | 0.00000175 |
|  | *Saitoella* | 0.00002870 | 0.00002800 |
|  | *Saprochaete* | 0.00000401 | 0.00000204 |
|  | *Schizosaccharomyces* | 0.00000331 | 0.00000266 |
|  | *Scutellospora* | 0.00002250 | 0.00001970 |
|  | *Serendipita* | 0.00000144 | 0.00000420 |
|  | *Smittium* | 0.00001070 | 0.00001320 |
|  | *Sparassis* | 0.00000301 | 0.00000443 |
|  | *Sphaerobolus* | 0.00000438 | 0.00000598 |
|  | *Sphaerulina* | 0.00000055 | 0.00000161 |
|  | *Sphagnurus* | 0.00000183 | 0.00000097 |
|  | *Spizellomyces* | 0.00005020 | 0.00003720 |
|  | *Sporisorium* | 0.00000275 | 0.00000090 |
|  | *Sporothrix* | 0.00000147 | 0.00000101 |
|  | *Steccherinum* | 0.00000021 | 0.00000117 |
|  | *Stereum* | 0.00000103 | 0.00000098 |
|  | *Sugiyamaella* | 0.00000874 | 0.00000980 |
|  | *Suillus* | 0.00000084 | 0.00000102 |
|  | *Syncephalastrum* | 0.00000913 | 0.00000885 |
|  | *Syncephalis* | 0.00000191 | 0.00000516 |
|  | *Talaromyces* | 0.00002180 | 0.00002760 |
|  | *Thamnidium* | 0.00000062 | 0.00000109 |
|  | *Thamnocephalis* | 0.00000909 | 0.00000658 |
|  | *Thelohania* | 0.00016496 | 0.00016220 |
|  | *Tilletia* | 0.00023006 | 0.00024399 |
|  | *Tilletiaria* | 0.00000131 | 0.00000025 |
|  | *Tilletiopsis* | 0.00000179 | 0.00000066 |
|  | *Torrubiella* | 0.00000113 | 0.00000043 |
|  | *Tortispora* | 0.00000106 | 0.00000178 |
|  | *Trametes* | 0.00012036 | 0.00012541 |
|  | *Tremella* | 0.00000343 | 0.00000349 |
|  | *Trichoderma* | 0.00000373 | 0.00000243 |
|  | *Tricholomella* | 0.00017760 | 0.00014764 |
|  | *Trichomonascus* | 0.00002620 | 0.00002630 |
|  | *Trichosporon* | 0.00000102 | 0.00000057 |
|  | *Tuber* | 0.00002330 | 0.00002410 |
|  | *Tulasnella* | 0.00001100 | 0.00000830 |
|  | *Tulosesus* | 0.00016898 | 0.00016780 |
|  | *Umbelopsis* | 0.00002740 | 0.00001710 |
|  | *Ustilago* | 0.00001870 | 0.00001680 |
|  | *Vanrija* | 0.00000143 | 0.00000163 |
|  | *Wallemia* | 0.00000182 | 0.00000313 |
|  | *Wickerhamomyces* | 0.00000141 | 0.00000153 |
|  | *Zancudomyces* | 0.00000721 | 0.00000455 |
| Viruses | Alphabaculovirus | 0.00000125 | 0.00000214 |
|  | Alphadintovirus | 0.00019534 | 0.00017879 |
|  | Alphaentomopoxvirus | 0.00000198 | 0.00000206 |
|  | Alpharetrovirus | 0.00000676 | 0.00000728 |
|  | Avipoxvirus | 0.00001140 | 0.00001160 |
|  | Betabaculovirus | 0.00000288 | 0.00000201 |
|  | Betaentomopoxvirus | 0.00000053 | 0.00000075 |
|  | Betaretrovirus | 0.00009080 | 0.00002920 |
|  | Bracovirus | 0.00004700 | 0.00004150 |
|  | Errantivirus | 0.00000427 | 0.00000192 |
|  | Gammaretrovirus | 0.00001680 | 0.00010804 |
|  | Ichnovirus | 0.00000692 | 0.00000678 |
|  | Iridovirus | 0.00000029 | 0.00000108 |
|  | Lymphocystivirus | 0.00002340 | 0.00003150 |
|  | Manticavirus | 0.00000095 | 0.00000060 |
|  | Orthopoxvirus | 0.00000121 | 0.00000101 |
|  | Pandoravirus | 0.00002200 | 0.00001940 |
|  | Proboscivirus | 0.00001790 | 0.00001470 |
|  | Quaranjavirus | 0.00017275 | 0.00015528 |
|  | Yatapoxvirus | 0.00000303 | 0.00000396 |
