# Supplementary material for: Metagenome reveals the midgut microbial community of Haemaphysalis qinghaiensis ticks collected from yaks and Tibetan sheep
Source: Parasit Vectors. 2024 Aug 31;17:370. doi: 10.1186/s13071-024-06442-y (PMC11366167; doi:10.1186/s13071-024-06442-y)
Supplement: Supplementary file 3 — Additional file 3: Table S3. Relative abundance of other than the top 25 common bacterial species in the two groups of Haemaphysalis qinghaiensis. [file 13071_2024_6442_MOESM3_ESM.docx]

**Additional File 3: Table S3.** Relative abundance of other than the top 25 common bacterial species in the two groups of *Haemaphysalis qinghaiensis*.

| Bacteria | Abundance (%) | |
| --- | --- | --- |
|  | Hq. C | Hq. S |
| *Acidobacteria bacterium* | 0.00001130 | 0.00000889 |
| *Acidobacteria bacterium* ADurb. Bin340 | 0.00000095 | 0.00000151 |
| *Acidobacterium capsulatum* | 0.00000024 | 0.00000139 |
| *Acidovorax avenae* | 0.00000267 | 0.00000085 |
| *Acinetobacter bereziniae* | 0.00001690 | 0.00001820 |
| *Acinetobacter pittii* | 0.00000652 | 0.00000368 |
| *Actinobacteria bacterium* | 0.00000135 | 0.00000149 |
| *Actinobacteria bacterium* OK006 | 0.00000138 | 0.00000016 |
| *Actinoplanes* sp. TRM66264-DLM | 0.00000197 | 0.00000063 |
| *Actinopolymorpha alba* | 0.00000079 | 0.00000477 |
| *Aequorivita* sp. 609 | 0.00000098 | 0.00000246 |
| *Aeromicrobium phragmitis* | 0.00000851 | 0.00000678 |
| *Aeromonas cavernicola* | 0.00001620 | 0.00001300 |
| *Aeromonas veronii* | 0.00003240 | 0.00003080 |
| *Alcanivorax profundi* | 0.00001460 | 0.00001430 |
| *Algoriphagus lacus* | 0.00000083 | 0.00000118 |
| *Alicyclobacillus pomorum* | 0.00000342 | 0.00000341 |
| *Aliidongia dinghuensis* | 0.00040239 | 0.00042700 |
| *Alkalispirillum mobile* | 0.00000140 | 0.00000222 |
| *Alphaproteobacteria bacterium* | 0.00002290 | 0.00002340 |
| *Alteriqipengyuania lutimaris* | 0.00000044 | 0.00000166 |
| *Amycolatopsis* sp. BJA-103 | 0.00000297 | 0.00000063 |
| *Anabaena* sp. MDT14b | 0.00000090 | 0.00000107 |
| *Anaerolineaceae bacterium* oral taxon 439 | 0.00000032 | 0.00000092 |
| *Anaerolineae bacterium* | 0.00000580 | 0.00000411 |
| *Anaerolineales bacterium* | 0.00000133 | 0.00000126 |
| *Anaerosalibacter bizertensis* | 0.00000103 | 0.00000033 |
| *Aquifex* sp. | 0.00063479 | 0.00058146 |
| *Arsenophonus* sp. ENCA | 0.00000318 | 0.00000771 |
| *Aureimonas endophytica* | 0.00003330 | 0.00003510 |
| *Bacilli bacterium* | 0.00000246 | 0.00000201 |
| *Bacillus cereus* | 0.00000191 | 0.00000116 |
| *Bacillus obstructivus* | 0.00002870 | 0.00001300 |
| *Bacillus pseudomycoides* | 0.00000123 | 0.00000047 |
| *Bacillus* sp. SRB_331 | 0.00001300 | 0.00001400 |
| *Bacillus thuringiensis* | 0.00001390 | 0.00002050 |
| *Bacillus yapensis* | 0.00000551 | 0.00000031 |
| bacterium | 0.00002090 | 0.00001570 |
| bacterium (*Candidatus* Blackallbacteria) CG18_big_fil_WC_8_21_14_2_50_49_26 | 0.00000768 | 0.00000604 |
| bacterium BMS3Abin12 | 0.00000019 | 0.00000073 |
| bacterium endosymbiont of *Escarpia laminata* | 0.00019261 | 0.00017887 |
| bacterium HR19 | 0.00000175 | 0.00000212 |
| bacterium TMED198 | 0.00000068 | 0.00000086 |
| *Bacteroidetes bacterium* | 0.00002370 | 0.00003010 |
| *Bacteroidetes bacterium* 4572_77 | 0.00020619 | 0.00018274 |
| *Borreliella garinii* | 0.00001650 | 0.00002250 |
| *Bowmanella* sp. Y26 | 0.00000404 | 0.00000100 |
| *Brachybacterium sacelli* | 0.00000109 | 0.00000207 |
| *Brachyspira hampsonii* | 0.00049746 | 0.00037089 |
| *Bradyrhizobium* sp. AUGA SZCCT0182 | 0.00000224 | 0.00000349 |
| *Bradyrhizobium* sp. RD5-C2 | 0.00000168 | 0.00000319 |
| *Brevibacillus* sp. MMS20-4M-10-Y | 0.00000165 | 0.00000148 |
| *Burkholderia cenocepacia* | 0.00000425 | 0.00000761 |
| *Burkholderia pseudomallei* | 0.00000143 | 0.00000092 |
| *Butyricimonas synergistica* | 0.00000035 | 0.00000066 |
| *Caedimonadaceae bacterium* | 0.00041364 | 0.00042901 |
| *Caldilineae bacterium* | 0.00000099 | 0.00000259 |
| *Caldiserica bacterium* CG_4_8_14_3_um_filter_35_18 | 0.00000042 | 0.00000110 |
| *Campylobacter coli* | 0.00013034 | 0.00019803 |
| *Campylobacter fetus* | 0.00004680 | 0.00004780 |
| *Campylobacter jejuni* | 0.00001240 | 0.00000493 |
| *Candidatus* Dadabacteria bacterium | 0.00000040 | 0.00000096 |
| *Candidatus* Enterovibrio escacola | 0.00001360 | 0.00001270 |
| *Candidatus* Eremiobacteraeota bacterium | 0.00000107 | 0.00000203 |
| *Candidatus* Erwinia dacicola | 0.00000977 | 0.00001220 |
| *Candidatus* Hodgkinia cicadicola | 0.00057309 | 0.00033850 |
| *Candidatus* Magnetomorum sp. HK-1 | 0.00000165 | 0.00000067 |
| *Candidatus* Marinimicrobia bacterium | 0.00000120 | 0.00000179 |
| *Candidatus* Nephrothrix sp. EaCA | 0.00011858 | 0.00009370 |
| *Candidatus* Pacebacteria bacterium CG10_big_fil_rev_8_21_14_0_10_42_12 | 0.00000093 | 0.00000124 |
| *Candidatus* Parcubacteria bacterium | 0.00000140 | 0.00000179 |
| *Candidatus* Regiella insecticola | 0.00004220 | 0.00004830 |
| *Candidatus* Rokubacteria bacterium | 0.00000055 | 0.00000130 |
| *Capsulimonas corticalis* | 0.00000038 | 0.00000164 |
| *Carnobacterium maltaromaticum* | 0.00012479 | 0.00010722 |
| *Catellatospora methionotrophica* | 0.00000290 | 0.00000132 |
| *Catellatospora* sp. IY07-71 | 0.00000107 | 0.00000068 |
| *Cecembia lonarensis* | 0.00000154 | 0.00000037 |
| *Cellulomonas hominis* | 0.00000097 | 0.00000111 |
| *Cellulomonas* sp. HLT2-17 | 0.00000112 | 0.00000129 |
| *Chitinophaga agrisoli* | 0.00001010 | 0.00001280 |
| *Chlamydia abortus* | 0.00001690 | 0.00001310 |
| *Chlamydia trachomatis* | 0.00000212 | 0.00000220 |
| *Chlorobium* sp. KB01 | 0.00000100 | 0.00000116 |
| *Chloroflexi bacterium* | 0.00000883 | 0.00000882 |
| *Chloroflexi bacterium* 13_1_20CM_50_12 | 0.00000440 | 0.00000357 |
| *Chloroflexi bacterium* HGW-Chloroflexi-1 | 0.00000080 | 0.00000136 |
| *Chloroflexi bacterium* HGW-Chloroflexi-10 | 0.00000041 | 0.00000077 |
| *Chloroflexi bacterium* RBG_13_50_21 | 0.00000052 | 0.00000136 |
| *Christensenella hongkongensis* | 0.00000118 | 0.00000063 |
| *Chryseobacterium* sp. RR2-3-20 | 0.00000510 | 0.00000309 |
| *Citrobacter freundii* | 0.00013982 | 0.00014238 |
| *Clostridioides difficile* | 0.00009070 | 0.00033601 |
| *Clostridium butyricum* | 0.00000188 | 0.00000051 |
| *Clostridium perfringens* | 0.00000283 | 0.00000073 |
| *Clostridium* sp. chh4-2 | 0.00001120 | 0.00000787 |
| *Cohnella* sp. GbtcB17 | 0.00000161 | 0.00000051 |
| *Comamonas* sp. CAH-2 | 0.00000102 | 0.00000169 |
| *Corynebacterium diphtheriae* | 0.00007330 | 0.00004350 |
| *Coxiella burnetii* | 0.00006700 | 0.00011212 |
| *Coxiella* endosymbiont of *Amblyomma americanum* | 0.00000651 | 0.00001140 |
| *Coxiella* endosymbiont of *Amblyomma nuttalli* | 0.00000344 | 0.00000630 |
| *Coxiella* endosymbiont of *Amblyomma sculptum* | 0.00000804 | 0.00001230 |
| *Coxiella* endosymbiont of *Rhipicephalus microplus* | 0.00002200 | 0.00003470 |
| *Coxiella* sp. RIFCSPHIGHO2_12_FULL_44_14 | 0.00000126 | 0.00000383 |
| *Coxiella* -like endosymbiont | 0.00001310 | 0.00001950 |
| *Crocosphaera subtropica* | 0.00000804 | 0.00000746 |
| *Crocosphaera watsonii* | 0.00001780 | 0.00001950 |
| *Curtobacterium* sp. ME26 | 0.00001160 | 0.00000850 |
| cyanobacterium TDX16 | 0.00000078 | 0.00000075 |
| *Cyanothece* sp. BG0011 | 0.00000609 | 0.00000819 |
| *Cycloclasticus* sp. symbiont of *Poecilosclerida* sp. M | 0.00007660 | 0.00007220 |
| *Deltaproteobacteria bacterium* | 0.00042632 | 0.00040090 |
| *Deltaproteobacteria bacterium* RIFOXYD12_FULL_50_9 | 0.00000155 | 0.00000186 |
| *Derxia lacustris* | 0.00000262 | 0.00000116 |
| *Desulfobacteraceae bacterium* 4484_190.1 | 0.00000108 | 0.00000026 |
| *Desulfocapsa* sp. | 0.00024692 | 0.00025452 |
| *Desulfosediminicola flagellatus* | 0.00000235 | 0.00000196 |
| *Devosia* sp. 66-22 | 0.00000032 | 0.00000090 |
| *Dongshaea marina* | 0.00000037 | 0.00000094 |
| *Eggerthia catenaformis* | 0.00010474 | 0.00010398 |
| endosymbiont of *Seepiophila jonesi* | 0.00001770 | 0.00001780 |
| endosymbiont of *Tevnia jerichonana* | 0.00003690 | 0.00003310 |
| *Endozoicomonas atrinae* | 0.00000226 | 0.00000023 |
| *Endozoicomonas elysicola* | 0.00000186 | 0.00000089 |
| *Endozoicomonas numazuensis* | 0.00000017 | 0.00000079 |
| *Enhygromyxa salina* | 0.00000178 | 0.00000153 |
| *Enterobacter cloacae* complex sp. 2DZ2F20B | 0.00042261 | 0.00037575 |
| *Enterobacter cloacae* complex sp. 2DZ2F2B | 0.00014052 | 0.00013002 |
| *Enterobacter cloacae* complex sp. 4DZ3-17B2 | 0.00007710 | 0.00005940 |
| *Enterobacter cloacae* complex sp. GF14B | 0.00000715 | 0.00000873 |
| *Enterobacter hormaechei* | 0.00002490 | 0.00000167 |
| *Enterobacter roggenkampii* | 0.00000270 | 0.00000088 |
| *Enterococcus casseliflavus* | 0.00007800 | 0.00008050 |
| *Epulopiscium* sp. AS2M-Bin002 | 0.00003300 | 0.00003340 |
| *Epulopiscium* sp. Nele67-Bin001 | 0.00010773 | 0.00009640 |
| *Epulopiscium* sp. Nele67-Bin004 | 0.00003660 | 0.00003500 |
| *Epulopiscium* sp. SCG-B11WGA-EpuloA1 | 0.00000100 | 0.00000225 |
| *Firmicutes bacterium* ADurb.Bin506 | 0.00000077 | 0.00000128 |
| *Flammeovirga kamogawensis* | 0.00000092 | 0.00000012 |
| *Flavobacteriales bacterium* | 0.00018840 | 0.00015449 |
| *Flavobacterium* johnsoniae | 0.00000689 | 0.00000356 |
| *Flavobacterium* salilacus | 0.00000113 | 0.00000036 |
| *Flavobacterium* sp. | 0.00001390 | 0.00001450 |
| *Flavobacterium* sp. JRM | 0.00015781 | 0.00016606 |
| *Flavobacterium* sp. JXAS1 | 0.00002320 | 0.00001610 |
| *Fontimonas thermophila* | 0.00000165 | 0.00000079 |
| *Francisella tularensis* | 0.00000241 | 0.00000535 |
| *Fusobacterium necrophorum* | 0.00000173 | 0.00000288 |
| *Gammaproteobacteria bacterium* 2W06 | 0.00003560 | 0.00003140 |
| *Gammaproteobacteria bacterium* CG_4_10_14_0_8_um_filter_38_16 | 0.00000136 | 0.00000065 |
| *Gammaproteobacteria bacterium* RIFCSPHIGHO2_12_FULL_38_11 | 0.00000045 | 0.00000100 |
| *Gemmatimonadetes bacterium* 13_1_40CM_69_22 | 0.00000175 | 0.00000160 |
| *Gloeocapsa* sp. DLM2.Bin57 | 0.00000193 | 0.00000307 |
| *Gloeothece citriformis* | 0.00000942 | 0.00001120 |
| *Glycocaulis profundi* | 0.00000436 | 0.00000439 |
| *Haemophilus influenzae* | 0.00000083 | 0.00000026 |
| *Hahella* sp. CCB-MM4 | 0.00000452 | 0.00001020 |
| *Haliangium ochraceum* | 0.00000202 | 0.00000192 |
| *Haloferula luteola* | 0.00000033 | 0.00000125 |
| *Halomonas xianhensis* | 0.00000122 | 0.00000261 |
| *Halopseudomonas pelagia* | 0.00000550 | 0.00000220 |
| *Halosaccharopolyspora lacisalsi* | 0.00000235 | 0.00000149 |
| *Helicobacter pylori* | 0.00000102 | 0.00000145 |
| *Herbaspirillum* sp. RU 5E | 0.00000271 | 0.00000258 |
| *Hydrocarboniphaga daqingensis* | 0.00000072 | 0.00000103 |
| *Jannaschia seosinensis* | 0.00000206 | 0.00000168 |
| *Ketobacter alkanivorans* | 0.00000265 | 0.00000268 |
| *Kitasatospora herbaricolor* | 0.00000158 | 0.00000194 |
| *Klebsiella aerogenes* | 0.00000083 | 0.00000132 |
| *Ktedonobacter* sp. SOSP1-85 | 0.00000250 | 0.00000232 |
| *Labedella endophytica* | 0.00001430 | 0.00001060 |
| *Lacticaseibacillus paracasei* | 0.00000332 | 0.00000158 |
| *Lactiplantibacillus plantarum* | 0.00000020 | 0.00000111 |
| *Legionella nagasakiensis* | 0.00001880 | 0.00001500 |
| *Leifsonia aquatica* | 0.00000220 | 0.00000197 |
| *Lewinella lacunae* | 0.00000031 | 0.00000147 |
| *Listeria monocytogenes* | 0.00008680 | 0.00011072 |
| *Listeria seeligeri* | 0.00008590 | 0.00007710 |
| *Lujinxingia litoralis* | 0.00000074 | 0.00000176 |
| *Macrococcus caseolyticus* | 0.00000434 | 0.00000174 |
| *Marinagarivorans* sp. GE09 | 0.00000055 | 0.00000235 |
| marine bacterium AO1-C | 0.00000018 | 0.00000086 |
| *Marinifilum breve* | 0.00005780 | 0.00005500 |
| *Marinococcus halophilus* | 0.00000056 | 0.00000081 |
| *Massilia plicata* | 0.00000083 | 0.00000237 |
| *Meiothermus hypogaeus* | 0.00000042 | 0.00000201 |
| *Membranicola marinus* | 0.00000192 | 0.00000209 |
| *Methylotenera* sp. | 0.00000092 | 0.00000175 |
| *Microbacterium arborescens* | 0.00004390 | 0.00004590 |
| *Microbacterium esteraromaticum* | 0.00002630 | 0.00002500 |
| *Microbacterium hydrothermale* | 0.00000170 | 0.00000269 |
| *Microcystis aeruginosa* | 0.00001200 | 0.00000854 |
| *Micromonospora* sp. RP3T | 0.00006360 | 0.00005400 |
| *Minicystis rosea* | 0.00000173 | 0.00000156 |
| *Moorena bouillonii* | 0.00000076 | 0.00000144 |
| *Morganella morganii* | 0.00004400 | 0.00003380 |
| *Mycoavidus* sp. B2-EB | 0.00000166 | 0.00000316 |
| *Mycobacterium colombiense* | 0.00000082 | 0.00000104 |
| *Mycobacterium* sp. 852014-50255_SCH5639931 | 0.00058568 | 0.00053455 |
| *Mycobacterium tuberculosis* | 0.00007940 | 0.00005820 |
| *Mycolicibacterium malmesburyense* | 0.00015336 | 0.00015272 |
| *Myxococcales bacterium* | 0.00000368 | 0.00000377 |
| *Nannocystis exedens* | 0.00000701 | 0.00000580 |
| *Neisseria meningitidis* | 0.00002370 | 0.00002750 |
| *Neobacillus vireti* | 0.00000213 | 0.00000015 |
| *Nitratireductor aestuarii* | 0.00011465 | 0.00011905 |
| *Nitrosococcus halophilus* | 0.00000018 | 0.00000083 |
| *Nocardia otitidiscaviarum* | 0.00007640 | 0.00011149 |
| *Nocardiopsis alkaliphila* | 0.00000071 | 0.00000023 |
| *Oceanicoccus sagamiensis* | 0.00000074 | 0.00000024 |
| *Oceanicoccus* sp. KOV_DT_Chl | 0.00000253 | 0.00000131 |
| *Oceaniferula marina* | 0.00000068 | 0.00000024 |
| *Oceanimonas baumannii* | 0.00000259 | 0.00000141 |
| *Oceanospirillum multiglobuliferum* | 0.00008480 | 0.00007680 |
| *Paenibacillus lautus* | 0.00000230 | 0.00000046 |
| *Paenibacillus montanisoli* | 0.00000250 | 0.00000262 |
| *Paenibacillus* sp. HW567 | 0.00000076 | 0.00000116 |
| *Paenibacillus* sp. S3N08 | 0.00000068 | 0.00000113 |
| *Panacagrimonas perspica* | 0.00000047 | 0.00000296 |
| *Pantoea vagans* | 0.00000391 | 0.00000153 |
| *Paracoccus acridae* | 0.00000066 | 0.00000249 |
| *Pararhizobium haloflavum* | 0.00000160 | 0.00000177 |
| *Pararhodobacter zhoushanensis* | 0.00000180 | 0.00000062 |
| *Pasteurella multocida* | 0.00000216 | 0.00000176 |
| *Pedobacter* sp. HMF7056 | 0.00000378 | 0.00000309 |
| *Phenylobacterium* sp. SCN 70-31 | 0.00000078 | 0.00000130 |
| *Phycisphaerales bacterium* | 0.00000134 | 0.00000013 |
| *Planctomyces* sp. SH-PL62 | 0.00000257 | 0.00000244 |
| *Planctomycetaceae bacterium* | 0.00000141 | 0.00000281 |
| *Planctomycetes bacterium* | 0.00000159 | 0.00000163 |
| *Planctomycetes bacterium* B3_Pla | 0.00004920 | 0.00004660 |
| *Polaribacter* sp. 20A6 | 0.00001280 | 0.00001370 |
| *Polyangium fumosum* | 0.00000087 | 0.00000107 |
| *Prosthecobacter vanneervenii* | 0.00000150 | 0.00000057 |
| *Proteobacteria bacterium* | 0.00000352 | 0.00000590 |
| *Proteobacteria bacterium* CAG:495 | 0.00000108 | 0.00000034 |
| *Pseudoalteromonas profundi* | 0.00000125 | 0.00000156 |
| *Pseudoalteromonas rubra* | 0.00000222 | 0.00000120 |
| *Pseudobacteriovorax antillogorgiicola* | 0.00000136 | 0.00000129 |
| *Pseudomonas* sp. S60 | 0.00000111 | 0.00000035 |
| *Pseudomonas syringae* | 0.00014751 | 0.00013598 |
| *Pseudomonas taeanensis* | 0.00000050 | 0.00000161 |
| *Raoultella ornithinolytica* | 0.00000183 | 0.00000104 |
| *Reyranella* sp. CPCC 100927 | 0.00000089 | 0.00000099 |
| *Rhabdochromatium marinum* | 0.00003150 | 0.00002290 |
| *Rhodococcus* sp. 114MFTsu3.1 | 0.00005150 | 0.00004280 |
| *Richelia intracellularis* | 0.00000236 | 0.00000096 |
| *Rickettsia amblyommatis* | 0.00054527 | 0.00047660 |
| *Rickettsiaceae bacterium* | 0.00005350 | 0.00004720 |
| *Rickettsiales bacterium* | 0.00000164 | 0.00000125 |
| *Riemerella columbipharyngis* | 0.00000072 | 0.00000102 |
| *Rippkaea orientalis* | 0.00000078 | 0.00000093 |
| *Rubritalea squalenifaciens* | 0.00000128 | 0.00000081 |
| *Rubrivivax* sp. | 0.00000851 | 0.00000830 |
| *Saccharopolyspora erythraea* | 0.00000178 | 0.00000099 |
| *Saccharopolyspora rhizosphaerae* | 0.00000246 | 0.00000327 |
| *Salinivenus iranica* | 0.00000114 | 0.00000108 |
| *Salmonella enterica* | 0.00000041 | 0.00000271 |
| *Salmonella* sp. S146_54837 | 0.00015078 | 0.00012058 |
| *Salmonella* sp. S152_61454 | 0.00000100 | 0.00000068 |
| *Sandaracinaceae bacterium* | 0.00000158 | 0.00000090 |
| *Schaalia georgiae* | 0.00002260 | 0.00002050 |
| *Soehngenia saccharolytica* | 0.00000125 | 0.00000134 |
| *Solemya elarraichensis* gill symbiont | 0.00017643 | 0.00017962 |
| *Sorangiineae bacterium* NIC37A_2 | 0.00000172 | 0.00000070 |
| *Sorangium cellulosum* | 0.00000671 | 0.00000398 |
| *Sphingobacteriaceae bacterium* | 0.00013272 | 0.00013500 |
| *Sphingobacteriales bacterium* | 0.00000137 | 0.00000026 |
| *Sphingobium* sp. AS12 | 0.00000614 | 0.00000546 |
| *Sphingomonas* sp. UNC305MFCol5.2 | 0.00000145 | 0.00000184 |
| *Spirulina major* | 0.00000078 | 0.00000018 |
| *Sporolactobacillus* sp. THM7-4 | 0.00000020 | 0.00000133 |
| *Streptococcus mitis* | 0.00000311 | 0.00000246 |
| *Streptococcus pneumoniae* | 0.00025397 | 0.00024101 |
| *Streptococcus pyogenes* | 0.00006750 | 0.00005620 |
| *Streptococcus suis* | 0.00004680 | 0.00003660 |
| *Streptomyces aureoverticillatus* | 0.00000051 | 0.00000122 |
| *Streptomyces mutabilis* | 0.00000172 | 0.00000094 |
| *Streptomyces narbonensis* | 0.00000160 | 0.00000025 |
| *Streptomyces* sp. AV19 | 0.00000035 | 0.00000133 |
| *Tamlana* sp. s12 | 0.00000282 | 0.00000810 |
| *Terracidiphilus gabretensis* | 0.00000032 | 0.00000120 |
| *Thermosynechococcus vestitus* | 0.00000202 | 0.00000048 |
| *Thiohalocapsa* sp. ML1 | 0.00000162 | 0.00000176 |
| *Thiothrix caldifontis* | 0.00000105 | 0.00000057 |
| *Thiothrix* sp. | 0.00007970 | 0.00008190 |
| *Tissierella creatinini* | 0.00000058 | 0.00000165 |
| *Treponema* sp. CETP13 | 0.00000113 | 0.00000089 |
| *Trichococcus shcherbakoviae* | 0.00000124 | 0.00000094 |
| *Tuwongella immobilis* | 0.00000029 | 0.00000111 |
| uncultured bacterium | 0.00001530 | 0.00001590 |
| uncultured *Rubrobacteraceae bacterium* | 0.00000269 | 0.00000117 |
| uncultured *Segetibacter* sp. | 0.00000971 | 0.00000986 |
| uncultured spirochete | 0.00000062 | 0.00000020 |
| uncultured *Synechococcales cyanobacterium* | 0.00000373 | 0.00000216 |
| *Variovorax* sp. YR216 | 0.00000062 | 0.00000107 |
| *Varunaivibrio sulfuroxidans* | 0.00000087 | 0.00000221 |
| *Verrucomicrobia bacterium* | 0.00002200 | 0.00002120 |
| *Verrucomicrobia bacterium* ADurb.Bin345 | 0.00000298 | 0.00000168 |
| *Verrucomicrobia subdivision* 3 bacterium | 0.00000041 | 0.00000098 |
| *Vibrio aquaticus* | 0.00000212 | 0.00000115 |
| *Vibrio parahaemolyticus* | 0.00005060 | 0.00004070 |
| *Virgibacillus massiliensis* | 0.00001360 | 0.00001370 |
| *Vitiosangium* sp. GDMCC 1.1324 | 0.00000111 | 0.00000247 |
| *Wolbachia* endosymbiont of *Atemnus politus* | 0.00028519 | 0.00026283 |
| *Wolbachia* endosymbiont of *Callosobruchus chinensis* | 0.00008260 | 0.00008840 |
| *Wolbachia* endosymbiont of *Drosophila ananassae* | 0.00004750 | 0.00004290 |
| *Wolbachia* endosymbiont of *Mansonella perstans* | 0.00006530 | 0.00006690 |
| *Wolbachia pipientis* | 0.00011616 | 0.00011540 |
| *Xanthomonas citri* | 0.00024855 | 0.00025655 |
| *Xylella fastidiosa* | 0.00000207 | 0.00000083 |
| *Yersinia kristensenii* | 0.00000178 | 0.00000127 |
| *Zetaproteobacteria bacterium* | 0.00000276 | 0.00000367 |
| *Zooshikella* sp. WH53 | 0.00000264 | 0.00000276 |
| *Zymomonas mobilis* | 0.00000244 | 0.00000103 |
