# Supplementary material for: Metagenome reveals the midgut microbial community of Haemaphysalis qinghaiensis ticks collected from yaks and Tibetan sheep
Source: Parasit Vectors. 2024 Aug 31;17:370. doi: 10.1186/s13071-024-06442-y (PMC11366167; doi:10.1186/s13071-024-06442-y)
Supplement: Supplementary file 4 — Additional file 4: Table S4. Relative abundance of other than the top 10 common viral species in the two groups of Haemaphysalis qinghaiensis. [file 13071_2024_6442_MOESM4_ESM.docx]

**Additional File 4: Table S4.** Relative abundance of other than the top 10 common viral species in the two groups of *Haemaphysalis qinghaiensis*.

| Viruses | Abundance (%) | |
| --- | --- | --- |
|  | Hq. C | Hq. S |
| Acanthamoeba castellanii medusavirus | 0.00000229 | 0.00000218 |
| Anomala cuprea entomopoxvirus | 0.00000198 | 0.00000206 |
| Avian leukosis virus | 0.00000096 | 0.00000036 |
| Bovine retrovirus CH15 | 0.00000453 | 0.00000490 |
| Brazilian porcupinepox virus 1 | 0.00000172 | 0.00000016 |
| Camelpox virus | 0.00000121 | 0.00000101 |
| Campoletis sonorensis ichnovirus | 0.00000071 | 0.00000048 |
| Canarypox virus | 0.00000081 | 0.00000026 |
| Catopsilia pomona nucleopolyhedrovirus | 0.00000100 | 0.00000095 |
| Cheloniid poxvirus 1 | 0.00000164 | 0.00000170 |
| Choristoneura fumiferana granulovirus | 0.00000112 | 0.00000080 |
| Chrysodeixis includens nucleopolyhedrovirus | 0.00000025 | 0.00000119 |
| Cotesia sesamiae bracovirus | 0.00000081 | 0.00000103 |
| Cotesia vestalis bracovirus | 0.00000027 | 0.00000129 |
| Densovirinae sp. | 0.00000890 | 0.00000730 |
| Elephant endotheliotropic herpesvirus 3 | 0.00000590 | 0.00000393 |
| Elephant endotheliotropic herpesvirus 4 | 0.00000338 | 0.00000343 |
| Elephantid betaherpesvirus 1 | 0.00000618 | 0.00000334 |
| Galidia ERV | 0.00000831 | 0.00000592 |
| Hemigrapsus takanoi nimavirus | 0.00000125 | 0.00000071 |
| Hubei toti-like virus 24 | 0.00000195 | 0.00000241 |
| Human endogenous retrovirus | 0.00000234 | 0.00000591 |
| Human endogenous retrovirus K | 0.00000679 | 0.00001780 |
| Hyposoter didymator ichnovirus | 0.00000086 | 0.00000136 |
| Hyposoter fugitivus ichnovirus | 0.00000232 | 0.00000327 |
| Hypsugopox virus | 0.00000437 | 0.00000325 |
| Idiomarinaceae phage Phi1M2-2 | 0.00000466 | 0.00000449 |
| Invertebrate iridescent virus 31 | 0.00000029 | 0.00000108 |
| IRE_CTVM19-associated rhabdovirus | 0.00000084 | 0.00000048 |
| Lampyris noctiluca errantivirus 1 | 0.00000246 | 0.00000039 |
| Lone star tick densovirus 1 | 0.00000325 | 0.00000320 |
| Lymphocystis disease virus 4 | 0.00000470 | 0.00000639 |
| Magpiepox virus | 0.00000043 | 0.00000182 |
| Manly virus | 0.00000232 | 0.00000125 |
| Matsumuraeses phaseoli granulovirus | 0.00000057 | 0.00000121 |
| Mouse mammary tumor virus | 0.00001020 | 0.00000465 |
| Myotis myotis endogenous retrovirus | 0.00000282 | 0.00000100 |
| Mythimna separata entomopoxvirus | 0.00000053 | 0.00000075 |
| Pandoravirus macleodensis | 0.00001070 | 0.00000930 |
| Pandoravirus neocaledonia | 0.00000545 | 0.00000560 |
| Periparus ater parvoviridae sp. | 0.00000097 | 0.00000026 |
| Phascolarctid gammaherpesvirus 1 | 0.00000095 | 0.00000060 |
| Tanapox virus | 0.00000303 | 0.00000396 |
| Teiidae poxvirus 1 | 0.00000816 | 0.00000479 |
| Tranosema rostrale ichnovirus | 0.00000150 | 0.00000054 |
| Trichoplusia ni TED virus | 0.00000181 | 0.00000153 |
| Turkeypox virus | 0.00000092 | 0.00000065 |
| UR2 sarcoma virus | 0.00000226 | 0.00000183 |
| Vibrio phage pYD21-A | 0.00000218 | 0.00000324 |
| Wuhan insect virus 22 | 0.00000337 | 0.00000371 |
| Yasminevirus sp. GU-2018 | 0.00000124 | 0.00000110 |
