# Supplementary material for: Metagenome reveals the midgut microbial community of Haemaphysalis qinghaiensis ticks collected from yaks and Tibetan sheep
Source: Parasit Vectors. 2024 Aug 31;17:370. doi: 10.1186/s13071-024-06442-y (PMC11366167; doi:10.1186/s13071-024-06442-y)
Supplement: Supplementary file 5 — Additional file 5: Table S5. Relative abundance of the common eukaryotic species in the two groups of Haemaphysalis qinghaiensis. [file 13071_2024_6442_MOESM5_ESM.docx]

**Additional File 5: Table S5.** Relative abundance of the common eukaryotic species in the two groups of *Haemaphysalis qinghaiensis*.

| Eukaryota | Abundance (%) | |
| --- | --- | --- |
|  | Hq. C | Hq. S |
| *Absidia glauca* | 0.00001560 | 0.00001440 |
| *Absidia repens* | 0.00000541 | 0.00000831 |
| *Acaromyces ingoldii* | 0.00000264 | 0.00000126 |
| *Acaulospora morrowiae* | 0.00011333 | 0.00011036 |
| *Actinomortierella ambigua* | 0.00000841 | 0.00000573 |
| *Agaricus bisporus* | 0.00001110 | 0.00000851 |
| *Agrocybe pediades* | 0.00001020 | 0.00000457 |
| *Akanthomyces lecanii* | 0.00006560 | 0.00006920 |
| *Allomyces macrogynus* | 0.00000639 | 0.00000454 |
| *Alternaria alternata* | 0.00000192 | 0.00000078 |
| *Amanita brunnescens* | 0.00000710 | 0.00000593 |
| *Amanita inopinata* | 0.00000138 | 0.00000058 |
| *Amanita muscaria* | 0.00000320 | 0.00000400 |
| *Ambispora gerdemannii* | 0.00000487 | 0.00000345 |
| *Ambispora leptoticha* | 0.00010018 | 0.00008450 |
| *Amphiamblys* sp. WSBS2006 | 0.00000206 | 0.00000112 |
| *Anaeromyces robustus* | 0.00001350 | 0.00000840 |
| *Anncaliia algerae* | 0.00001020 | 0.00000722 |
| *Antrodiella citrinella* | 0.00000194 | 0.00000053 |
| *Apophysomyces ossiformis* | 0.00002070 | 0.00001660 |
| *Ascobolus immersus* | 0.00000299 | 0.00000328 |
| *Ascosphaera apis* | 0.00000339 | 0.00000288 |
| *Aspergillus calidoustus* | 0.00000291 | 0.00000263 |
| *Aspergillus fumigatiaffinis* | 0.00000026 | 0.00000124 |
| *Aspergillus fumigatus* | 0.00000327 | 0.00000387 |
| *Aspergillus wentii* | 0.00000135 | 0.00000064 |
| *Auriculariales* sp. MPI-PUGE-AT-0066 | 0.00000129 | 0.00000070 |
| *Basidiobolus meristosporus* | 0.00005610 | 0.00005850 |
| *Batrachochytrium dendrobatidis* | 0.00000544 | 0.00000645 |
| *Batrachochytrium salamandrivorans* | 0.00001660 | 0.00001470 |
| *Bifiguratus adelaidae* | 0.00000697 | 0.00000749 |
| *Bipolaris maydis* | 0.00000123 | 0.00000047 |
| *Blyttiomyces helicus* | 0.00000549 | 0.00000399 |
| *Boletus reticuloceps* | 0.00000028 | 0.00000122 |
| *Cadophora* sp. DSE1049 | 0.00000099 | 0.00000158 |
| *Calocera cornea* | 0.00000107 | 0.00000076 |
| *Calocera viscosa* | 0.00001710 | 0.00001080 |
| *Candida arabinofermentans* | 0.00000186 | 0.00000291 |
| *Candida auris* | 0.00000108 | 0.00000123 |
| *Candida metapsilosis* | 0.00000154 | 0.00000173 |
| *Candolleomyces aberdarensis* | 0.00000059 | 0.00000254 |
| *Cantharellus anzutake* | 0.00000106 | 0.00000189 |
| *Catenaria anguillulae* | 0.00001520 | 0.00002490 |
| *Caulochytrium protostelioides* | 0.00000493 | 0.00000459 |
| *Ceraceosorus bombacis* | 0.00000602 | 0.00000341 |
| *Ceratobasidium* sp. 370 | 0.00000371 | 0.00000184 |
| *Ceratobasidium* sp. 395 | 0.00000616 | 0.00000778 |
| *Ceratobasidium* sp. AG-Ba | 0.00008600 | 0.00007160 |
| *Ceratobasidium* sp. AG-I | 0.00000242 | 0.00000033 |
| *Ceratobasidium theobromae* | 0.00001580 | 0.00001410 |
| *Cercospora kikuchii* | 0.00001800 | 0.00001390 |
| *Cetraspora pellucida* | 0.00002770 | 0.00002500 |
| *Chaetomium globosum* | 0.00000425 | 0.00000795 |
| *Chalara longipes* | 0.00000027 | 0.00000182 |
| *Chlorociboria aeruginascens* | 0.00000276 | 0.00000117 |
| *Choanephora cucurbitarum* | 0.00003320 | 0.00003650 |
| *Choiromyces venosus* | 0.00000306 | 0.00000367 |
| *Chytriomyces confervae* | 0.00000643 | 0.00001050 |
| *Claroideoglomus candidum* | 0.00031411 | 0.00029155 |
| *Claviceps purpurea* | 0.00000140 | 0.00000346 |
| *Coemansia reversa* | 0.00000725 | 0.00000682 |
| *Coleophoma cylindrospora* | 0.00000141 | 0.00000027 |
| *Colletotrichum asianum* | 0.00000285 | 0.00000339 |
| *Conidiobolus coronatus* | 0.00000540 | 0.00000732 |
| *Coniochaeta ligniaria* | 0.00000125 | 0.00000024 |
| *Coniochaeta* sp. 2T2.1 | 0.00000145 | 0.00000061 |
| *Coprinopsis cinerea* | 0.00001620 | 0.00002230 |
| *Cronartium quercuum* | 0.00000336 | 0.00000269 |
| *Cryptococcus floricola* | 0.00000862 | 0.00000715 |
| *Cryptococcus wingfieldii* | 0.00000164 | 0.00000114 |
| *Cucumispora dikerogammari* | 0.00054522 | 0.00045158 |
| *Cylindrobasidium torrendii* | 0.00000196 | 0.00000196 |
| *Daedalea quercina* | 0.00000107 | 0.00000058 |
| *Dentipellis* sp. KUC8613 | 0.00000061 | 0.00000138 |
| *Dentiscutata erythropus* | 0.00006740 | 0.00006870 |
| *Dentiscutata heterogama* | 0.00003660 | 0.00003330 |
| *Dictyocoela muelleri* | 0.00054799 | 0.00054057 |
| *Dictyocoela roeselum* | 0.00000353 | 0.00000465 |
| *Dimargaris cristalligena* | 0.00001140 | 0.00000574 |
| *Dissophora globulifera* | 0.00000091 | 0.00000159 |
| *Diutina rugosa* | 0.00000040 | 0.00000096 |
| *Diversispora eburnea* | 0.00004090 | 0.00004060 |
| *Diversispora epigaea* | 0.00002070 | 0.00001540 |
| *Endogone* sp. FLAS-F59071 | 0.00001350 | 0.00001390 |
| *Enterospora canceri* | 0.00013031 | 0.00012237 |
| *Entomophthora muscae* | 0.00000539 | 0.00000555 |
| *Entomortierella beljakovae* | 0.00000749 | 0.00000986 |
| *Entomortierella parvispora* | 0.00000220 | 0.00000254 |
| *Erysiphe pulchra* | 0.00006210 | 0.00006490 |
| *Escovopsis weberi* | 0.00000557 | 0.00000310 |
| *Exidia glandulosa* | 0.00000063 | 0.00000100 |
| *Exophiala xenobiotica* | 0.00000050 | 0.00000079 |
| *Fistulina hepatica* | 0.00000123 | 0.00000047 |
| *Friedmanniomyces simplex* | 0.00000193 | 0.00000071 |
| *Fulvia fulva* | 0.00001220 | 0.00000867 |
| *Funneliformis caledonium* | 0.00000418 | 0.00000097 |
| *Funneliformis mosseae* | 0.00000581 | 0.00000638 |
| *Furculomyces boomerangus* | 0.00000066 | 0.00000079 |
| *Fusarium oxysporum* | 0.00004160 | 0.00003230 |
| *Gaeumannomyces tritici* | 0.00000083 | 0.00000118 |
| *Gamsiella multidivaricata* | 0.00000229 | 0.00000188 |
| *Ganoderma sinense* | 0.00000142 | 0.00000236 |
| *Geosiphon pyriformis* | 0.00003930 | 0.00003550 |
| *Geosmithia morbida* | 0.00000089 | 0.00000021 |
| *Geotrichum candidum* | 0.00000266 | 0.00000179 |
| *Gigaspora margarita* | 0.00001430 | 0.00001270 |
| *Gigaspora rosea* | 0.00001940 | 0.00001410 |
| *Gloeophyllum trabeum* | 0.00000223 | 0.00000061 |
| *Glomus cerebriforme* | 0.00000266 | 0.00000144 |
| *Gonapodya prolifera* | 0.00004480 | 0.00003720 |
| *Gryganskiella cystojenkinii* | 0.00000183 | 0.00000119 |
| *Gymnopilus dilepis* | 0.00002870 | 0.00002890 |
| *Hamiltosporidium tvaerminnensis* | 0.00001810 | 0.00001760 |
| *Haplosporangium gracile* | 0.00000337 | 0.00000272 |
| *Haplosporangium* sp. Z 11 | 0.00000190 | 0.00000090 |
| *Haplosporangium* sp. Z 27 | 0.00000294 | 0.00000593 |
| *Helotiales* sp. DMI_Dod_QoI | 0.00000144 | 0.00000023 |
| *Hepatospora eriocheir* | 0.00001170 | 0.00001000 |
| *Hericium alpestre* | 0.00000277 | 0.00000244 |
| *Hermanssonia centrifuga* | 0.00021024 | 0.00019964 |
| *Hesseltinella vesiculosa* | 0.00000315 | 0.00000392 |
| *Hypsizygus marmoreus* | 0.00000085 | 0.00000182 |
| *Jimgerdemannia flammicorona* | 0.00001060 | 0.00001120 |
| *Kazachstania africana* | 0.00000092 | 0.00000157 |
| *Kazachstania exigua* | 0.00000407 | 0.00000631 |
| *Kluyveromyces lactis* | 0.00000148 | 0.00000061 |
| *Kluyveromyces marxianus* | 0.00000078 | 0.00000111 |
| *Kuraishia capsulata* | 0.00000110 | 0.00000023 |
| *Kwoniella shandongensis* | 0.00002450 | 0.00002900 |
| *Laccaria amethystina* | 0.00000428 | 0.00000298 |
| *Laetiporus sulphureus* | 0.00000124 | 0.00000196 |
| *Lasallia pustulata* | 0.00000390 | 0.00000346 |
| *Lentinula edodes* | 0.00002500 | 0.00002690 |
| *Leucosporidium creatinivorum* | 0.00000060 | 0.00000114 |
| *Lichtheimia corymbifera* | 0.00003310 | 0.00002360 |
| *Lichtheimia ramosa* | 0.00001030 | 0.00001090 |
| *Linderina pennispora* | 0.00000850 | 0.00000554 |
| *Linnemannia elongata* | 0.00000171 | 0.00000152 |
| *Linnemannia gamsii* | 0.00000215 | 0.00000285 |
| *Lipomyces starkeyi* | 0.00000320 | 0.00000121 |
| *Lobosporangium transversale* | 0.00000313 | 0.00000089 |
| *Lophiotrema nucula* | 0.00000192 | 0.00000125 |
| *Macrophomina phaseolina* | 0.00001330 | 0.00001690 |
| *Malassezia vespertilionis* | 0.00000242 | 0.00000139 |
| *Melanogaster broomeanus* | 0.00000109 | 0.00000052 |
| *Melanopsichium pennsylvanicum* | 0.00000303 | 0.00000081 |
| *Metarhizium rileyi* | 0.00000055 | 0.00000158 |
| *Metarhizium robertsii* | 0.00002260 | 0.00002080 |
| *Microbotryum saponariae* | 0.00000237 | 0.00000184 |
| *Mixia osmundae* | 0.00000046 | 0.00000227 |
| *Monilinia vaccinii-corymbosi* | 0.00000171 | 0.00000102 |
| *Monosporascus* sp. 5C6A | 0.00001450 | 0.00001590 |
| *Morchella conica* | 0.00000090 | 0.00000120 |
| *Morchella sextelata* | 0.00000908 | 0.00001020 |
| *Mortierella alpina* | 0.00000258 | 0.00000275 |
| *Mortierella antarctica* | 0.00000156 | 0.00000089 |
| *Mortierella polycephala* | 0.00000066 | 0.00000140 |
| *Mortierella* sp. AD032 | 0.00000070 | 0.00000110 |
| *Mortierella* sp. GBA30 | 0.00000050 | 0.00000134 |
| *Mortierella* sp. GBA39 | 0.00000279 | 0.00000144 |
| *Mortierella* sp. GBA43 | 0.00000722 | 0.00000549 |
| *Mucidula mucida* | 0.00000133 | 0.00000098 |
| *Mucor ambiguus* | 0.00000077 | 0.00000037 |
| *Mucor circinatus* | 0.00034917 | 0.00026230 |
| *Mucor circinelloides* | 0.00000430 | 0.00000625 |
| *Mucor plumbeus* | 0.00000777 | 0.00000311 |
| *Mucor saturninus* | 0.00009540 | 0.00009060 |
| *Mycena galopus* | 0.00000035 | 0.00000099 |
| *Mycena sanguinolenta* | 0.00000149 | 0.00000182 |
| *Myriangium duriaei* | 0.00000037 | 0.00000107 |
| *Mytilinidion resinicola* | 0.00000729 | 0.00000683 |
| *Naganishia liquefaciens* | 0.00000099 | 0.00000095 |
| *Nannizzia gypsea* | 0.00000478 | 0.00000630 |
| *Neocallimastix* sp. JGI-2020a | 0.00000879 | 0.00000779 |
| *Neolecta irregularis* | 0.00000222 | 0.00000192 |
| *Nosema apis* | 0.00002160 | 0.00002110 |
| *Nosema granulosis* | 0.00048082 | 0.00046425 |
| *Ogataea haglerorum* | 0.00000226 | 0.00000344 |
| *Olpidium bornovanus* | 0.00000572 | 0.00000736 |
| *Ophiostoma piceae* | 0.00000311 | 0.00000171 |
| *Orpinomyces* sp. OUS1 | 0.00000118 | 0.00000224 |
| *Paraglomus brasilianum* | 0.00000101 | 0.00000179 |
| *Paraglomus occultum* | 0.00007240 | 0.00007420 |
| *Paramicrosporidium saccamoebae* | 0.00000309 | 0.00000434 |
| *Parasitella parasitica* | 0.00002010 | 0.00001900 |
| *Patellaria atrata* | 0.00000195 | 0.00000093 |
| *Penicillium antarcticum* | 0.00000027 | 0.00000179 |
| *Penicillium brasilianum* | 0.00000118 | 0.00000238 |
| *Penicillium italicum* | 0.00000044 | 0.00000182 |
| *Penicillium nalgiovense* | 0.00000240 | 0.00000288 |
| *Penicillium nordicum* | 0.00006540 | 0.00006780 |
| *Phanerochaete sordida* | 0.00000190 | 0.00000119 |
| *Phycomyces blakesleeanus* | 0.00001250 | 0.00001050 |
| *Piptocephalis cylindrospora* | 0.00000167 | 0.00000126 |
| *Piromyces finnis* | 0.00001670 | 0.00002590 |
| *Piromyces* sp. E2 | 0.00000264 | 0.00000280 |
| *Pneumocystis oryctolagi* | 0.00000068 | 0.00000123 |
| *Podila clonocystis* | 0.00001120 | 0.00001330 |
| *Podila horticola* | 0.00000138 | 0.00000613 |
| *Podila verticillata* | 0.00000034 | 0.00000097 |
| *Polyplosphaeria fusca* | 0.00000072 | 0.00000103 |
| *Powellomyces hirtus* | 0.00000681 | 0.00001210 |
| *Pseudogymnoascus* sp. VKM F-4519 (FW-2642) | 0.00020745 | 0.00016618 |
| *Psilocybe cyanescens* | 0.00000168 | 0.00000032 |
| *Pterula gracilis* | 0.00000364 | 0.00000133 |
| *Puccinia coronata* | 0.00001630 | 0.00001870 |
| *Puccinia graminis* | 0.00003820 | 0.00002490 |
| *Puccinia sorghi* | 0.00000119 | 0.00000260 |
| *Puccinia striiformis* | 0.00006490 | 0.00006580 |
| *Puccinia triticina* | 0.00000512 | 0.00000308 |
| *Pyronema omphalodes* | 0.00000097 | 0.00000037 |
| *Pyrrhoderma noxium* | 0.00000105 | 0.00000025 |
| *Racocetra fulgida* | 0.00000126 | 0.00000315 |
| *Rhizoclosmatium globosum* | 0.00000756 | 0.00000268 |
| *Rhizoctonia solani* | 0.00003690 | 0.00004620 |
| *Rhizophagus clarus* | 0.00019449 | 0.00016459 |
| *Rhizophagus irregularis* | 0.00081776 | 0.00081617 |
| *Rhizophagus* sp. MUCL 43196 | 0.00000170 | 0.00000032 |
| *Rhizopus arrhizus* | 0.00022796 | 0.00020394 |
| *Rhizopus delemar* | 0.00004030 | 0.00004170 |
| *Rhizopus microsporus* | 0.00000540 | 0.00000368 |
| *Rhizopus stolonifer* | 0.00000165 | 0.00000125 |
| *Rhodotorula diobovata* | 0.00000057 | 0.00000109 |
| *Rhodotorula graminis* | 0.00000056 | 0.00000161 |
| *Rhodotorula toruloides* | 0.00000054 | 0.00000219 |
| *Rozella allomycis* | 0.00002420 | 0.00002890 |
| *Russula ochroleuca* | 0.00000381 | 0.00000300 |
| *Saccharomycodes ludwigii* | 0.00000092 | 0.00000175 |
| *Saitoella complicata* | 0.00002870 | 0.00002800 |
| *Saprochaete ingens* | 0.00000401 | 0.00000204 |
| *Schizosaccharomyces octosporus* | 0.00000138 | 0.00000092 |
| *Schizosaccharomyces pombe* | 0.00000119 | 0.00000062 |
| *Scutellospora calospora* | 0.00002250 | 0.00001970 |
| *Serendipita vermifera* | 0.00000074 | 0.00000398 |
| *Smittium culicis* | 0.00000908 | 0.00001190 |
| *Sparassis crispa* | 0.00000301 | 0.00000443 |
| *Sphaerobolus stellatus* | 0.00000438 | 0.00000598 |
| *Sphaerulina musiva* | 0.00000055 | 0.00000161 |
| *Sphagnurus paluster* | 0.00000183 | 0.00000097 |
| *Spizellomyces punctatus* | 0.00000923 | 0.00000594 |
| *Spizellomyces* sp. palustris | 0.00001670 | 0.00001120 |
| *Sporisorium graminicola* | 0.00000065 | 0.00000062 |
| *Sporisorium scitamineum* | 0.00000210 | 0.00000029 |
| *Steccherinum ochraceum* | 0.00000021 | 0.00000117 |
| *Stereum hirsutum* | 0.00000103 | 0.00000098 |
| *Sugiyamaella lignohabitans* | 0.00000874 | 0.00000980 |
| *Suillus clintonianus* | 0.00000084 | 0.00000102 |
| *Syncephalastrum racemosum* | 0.00000913 | 0.00000885 |
| *Syncephalis pseudoplumigaleata* | 0.00000191 | 0.00000516 |
| *Synchytrium endobioticum* | 0.00103474 | 0.00106050 |
| *Synchytrium microbalum* | 0.00009870 | 0.00012046 |
| *Talaromyces cellulolyticus* | 0.00000895 | 0.00001010 |
| *Talaromyces stipitatus* | 0.00001290 | 0.00001750 |
| *Thamnidium elegans* | 0.00000062 | 0.00000109 |
| *Thamnocephalis sphaerospora* | 0.00000909 | 0.00000658 |
| *Thelohania contejeani* | 0.00016496 | 0.00016220 |
| *Tilletia caries* | 0.00000031 | 0.00000194 |
| *Tilletia indica* | 0.00002510 | 0.00001970 |
| *Tilletia walkeri* | 0.00000083 | 0.00000059 |
| *Tilletiaria anomala* | 0.00000131 | 0.00000025 |
| *Tilletiopsis washingtonensis* | 0.00000179 | 0.00000066 |
| *Torrubiella hemipterigena* | 0.00000113 | 0.00000043 |
| *Tortispora caseinolytica* | 0.00000106 | 0.00000178 |
| *Trametes cinnabarina* | 0.00000717 | 0.00001170 |
| *Trametes pubescens* | 0.00010039 | 0.00009990 |
| *Trametes versicolor* | 0.00000373 | 0.00000283 |
| *Tremella mesenterica* | 0.00000343 | 0.00000349 |
| *Trichoderma cornu-damae* | 0.00000204 | 0.00000174 |
| *Trichoderma harzianum* | 0.00000169 | 0.00000069 |
| *Tricholomella constricta* | 0.00017760 | 0.00014764 |
| *Trichomonascus ciferrii* | 0.00002620 | 0.00002630 |
| *Trichosporon asahii* | 0.00000102 | 0.00000057 |
| *Tuber aestivum* | 0.00002120 | 0.00002300 |
| *Tuber indicum* | 0.00000211 | 0.00000115 |
| *Tulasnella calospora* | 0.00000332 | 0.00000330 |
| *Tulasnella* sp. 403 | 0.00000053 | 0.00000084 |
| *Tulasnella* sp. 417 | 0.00000667 | 0.00000306 |
| *Tulosesus angulatus* | 0.00016898 | 0.00016780 |
| *Umbelopsis isabellina* | 0.00001020 | 0.00000817 |
| *Umbelopsis vinacea* | 0.00001590 | 0.00000820 |
| *Ustilago maydis* | 0.00001120 | 0.00000717 |
| *Ustilago trichophora* | 0.00000744 | 0.00000964 |
| *Vanrija humicola* | 0.00000143 | 0.00000163 |
| *Wickerhamomyces pijperi* | 0.00000141 | 0.00000153 |
| *Zancudomyces culisetae* | 0.00000721 | 0.00000455 |
