# Supplementary material for: Metagenome reveals the midgut microbial community of Haemaphysalis qinghaiensis ticks collected from yaks and Tibetan sheep
Source: Parasit Vectors. 2024 Aug 31;17:370. doi: 10.1186/s13071-024-06442-y (PMC11366167; doi:10.1186/s13071-024-06442-y)
Supplement: Supplementary file 6 — Additional file 6: Table S6. Relative abundance of the common archaean species in the two groups of Haemaphysalis qinghaiensis. [file 13071_2024_6442_MOESM6_ESM.docx]

**Additional File 6: Table S6.** Relative abundance of the common archaean species in the two groups of *Haemaphysalis qinghaiensis*.

| Archaea | Abundance (%) | |
| --- | --- | --- |
|  | Hq. C | Hq. S |
| archaeon GW2011_AR11 | 0.00000400 | 0.00000464 |
| *Candidatus* Bathyarchaeota archaeon | 0.00015837 | 0.00014583 |
| *Candidatus* Heimdallarchaeota archaeon B3_Heim | 0.00004170 | 0.00003560 |
| *Candidatus* Nezhaarchaeota archaeon WYZ-LMO8 | 0.00000212 | 0.00000183 |
| *Euryarchaeota archaeon* | 0.00000160 | 0.00000122 |
| *Halomicroarcula salinisoli* | 0.00000245 | 0.00000146 |
| *Methanosarcinales archaeon* | 0.00000592 | 0.00000433 |
| *Natronococcus* sp. LS1_42 | 0.00000065 | 0.00000062 |
| *Nitrosopumilus maritimus* | 0.00000113 | 0.00000027 |
| *Thaumarchaeota archaeon* | 0.00000356 | 0.00000297 |
| *Thermoplasmata archaeon* | 0.00001010 | 0.00001280 |
| *Thermoproteus* sp. AZ2 | 0.00000099 | 0.00000047 |
